# Multi-scale light microscopy/electron microscopy neuronal imaging from brain to synapse with a tissue clearing method, Sca*l*eSF

**DOI:** 10.1016/j.isci.2021.103601

**Published:** 2021-12-27

**Authors:** Takahiro Furuta, Kenta Yamauchi, Shinichiro Okamoto, Megumu Takahashi, Soichiro Kakuta, Yoko Ishida, Aya Takenaka, Atsushi Yoshida, Yasuo Uchiyama, Masato Koike, Kaoru Isa, Tadashi Isa, Hiroyuki Hioki

**Affiliations:** 1 Department of Oral Anatomy and Neurobiology, Graduate School of Dentistry, Osaka University, Suita, Osaka 565-0871, Japan; 2 Department of Neuroanatomy, Juntendo University Graduate School of Medicine, Bunkyo-Ku, Tokyo 113-8421, Japan; 3 Department of Cell Biology and Neuroscience, Juntendo University Graduate School of Medicine, Bunkyo-Ku, Tokyo 113-8421, Japan; 4 Advanced Research Institute for Health Sciences, Juntendo University, Bunkyo-Ku, Tokyo 113-8421, Japan; 5 Department of Neuroscience, Graduate School of Medicine, Kyoto University, Kyoto, Kyoto 606-8501, Japan; 6 Laboratory of Morphology and Image Analysis, Biomedical Research Core Facilities, Juntendo University Graduate School of Medicine, Bunkyo-Ku, Tokyo 113-8421, Japan; 7 Department of Cellular and Molecular Neuropathology, Juntendo University Graduate School of Medicine, Bunkyo-Ku, Tokyo 113-8421, Japan; 8 Institute for the Advanced Study of Human Biology (WPI-ASHBi), Kyoto University, Kyoto, Kyoto 606-8501, Japan; 9 Department of Multi-Scale Brain Structure Imaging, Juntendo University Graduate School of Medicine, Bunkyo-Ku, Tokyo 113-8421, Japan

**Keywords:** Imaging anatomy, Optical imaging, Neuroscience, Cell biology

## Abstract

The mammalian brain is organized over sizes that span several orders of magnitude, from synapses to the entire brain. Thus, a technique to visualize neural circuits across multiple spatial scales (multi-scale neuronal imaging) is vital for deciphering brain-wide connectivity. Here, we developed this technique by coupling successive light microscopy/electron microscopy (LM/EM) imaging with a glutaraldehyde-resistant tissue clearing method, Sca*l*eSF. Our multi-scale neuronal imaging incorporates (1) brain-wide macroscopic observation, (2) mesoscopic circuit mapping, (3) microscopic subcellular imaging, and (4) EM imaging of nanoscopic structures, allowing seamless integration of structural information from the brain to synapses. We applied this technique to three neural circuits of two different species, mouse striatofugal, mouse callosal, and marmoset corticostriatal projection systems, and succeeded in simultaneous interrogation of their circuit structure and synaptic connectivity in a targeted way. Our multi-scale neuronal imaging will significantly advance the understanding of brain-wide connectivity by expanding the scales of objects.

## Highlights

• Successive light microscopy/electron microscopy in optically cleared tissues
• Multi-scale neuronal imaging from the entire brain to individual synapses
• Simultaneous interrogation of neural circuit structure and synaptic connectivity
• Zooming-in to scarce synaptic contacts with the successive imaging

## Graphical abstract

**Figure:**
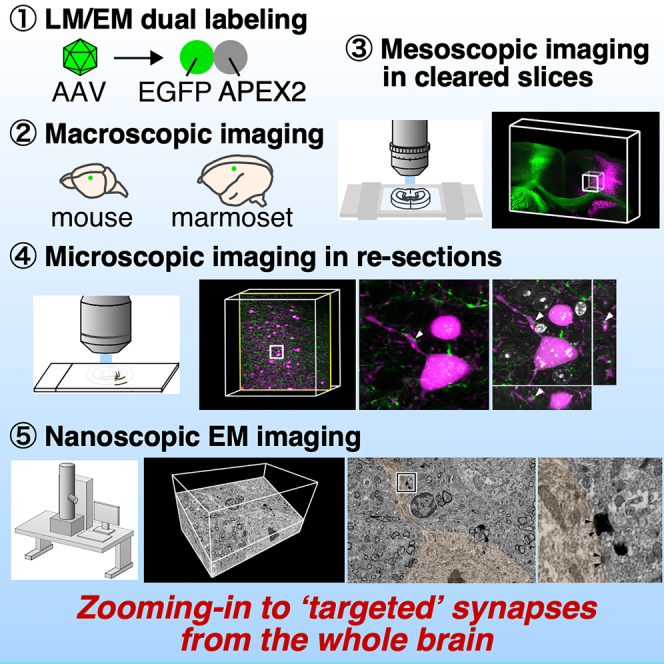

## Introduction

Connectomics, a description of a wiring diagram of the nervous system, is fundamental for understanding of how neural circuits process information and generate behavior (Lichtman and Sanes, 2008; Sporns et al., 2005). The mammalian brain contains a heterogeneous mixture of billions of neurons with trillions of synapses. Neurons elaborate highly specialized processes that can be over a meter in length for transmitting and receiving information, whereas synapses that connect neurons to one another are several hundred nanometers in size. Hence, the imaging scale required for deciphering brain-wide connectivity of mammalian brains is more than several orders of magnitude (Lichtman and Denk, 2011).

Electron microscopy (EM) provides an unparalleled resolution to trace nanometer-thin neuronal processes and identify a synapse unambiguously. Recent advances in volume EM, such as serial block-face scanning EM (SBF-SEM), focused ion beam milling and SEM (FIB-SEM), automated tape-collecting ultramicrotomy (ATUM) with SEM (ATUM-SEM), transmission-mode SEM (tSEM), and transmission EM (TEM) camera array (TEMCA), have enabled us to see ultrastructure within a significant volume of brain, opening up the possibility of assembling a connectome of a mammalian brain (Kornfeld and Denk, 2018; Kubota et al., 2018). However, current analysis has been limited to small volumes of tens to hundreds of micrometers in extent (Helmstaedter et al., 2013; Kasthuri et al., 2015; Motta et al., 2019; Schmidt et al., 2017).

Fluorescence light microscopy (LM) coupled with genetic labeling methods allows tracking of neuronal processes over long distances to assemble mesoscale connectomic maps for the mouse cerebral cortex and thalamus (Harris et al., 2019; Oh et al., 2014) and reconstruct individual neurons at subcellular resolution (Lin et al., 2018; Winnubst et al., 2019). Of particular note, tissue clearing techniques have drastically improved the depth-independent observation of biological samples with fluorescence LM, facilitating connectomic analysis with the scales from the macroscopic/brain to microscopic/subcellular level (Richardson and Lichtman, 2015; Ueda et al., 2020a, 2020b). However, despite its fundamental advances in spatial resolution (Schermelleh et al., 2010), the resolution of LM does not match the size of a synapse that defines neuronal connectivity. Indeed, axodendritic contacts identified by LM observation are only partially predictive of whether synapses are actually formed (da Costa and Martin, 2009; Holler et al., 2021). Importantly, a synapse, which consists of presynaptic membrane, postsynaptic membrane, and a synaptic cleft (chemical synapses) or a neuronal gap junction (electrical synapses), is defined by EM observation (Hama, 1959; Pease, 1953).

Here, we developed an imaging pipeline to decipher brain-wide connectivity across multiple spatial scales by coupling successive LM and EM (LM/EM) imaging with a tissue clearing technique (multi-scale LM/EM neuronal imaging). To achieve such imaging, we developed a glutaraldehyde (GA)-resistant tissue clearing technique, Sca*l*eSF. We further implemented LM/EM dual labeling that remained stable in the clearing protocol. We applied this technique to mouse striatofugal and marmoset corticostriatal projection systems and succeeded in simultaneous interrogation of their circuit structure and synaptic connectivity. In addition, we took advantage of the fact that our developed imaging system permitted LM imaging of substantial tissue volume at high-resolution followed by subsequent EM observation to capture scarce synaptic contacts with nanoscale resolution formed by brain-wide connectivity. We identified and tracked mouse callosal inputs onto parvalbumin (PV)-positive neocortical interneurons in a targeted way across multiple spatial scales.

## Results

### Multi-scale LM/EM neuronal imaging pipeline

**Figure 1:**
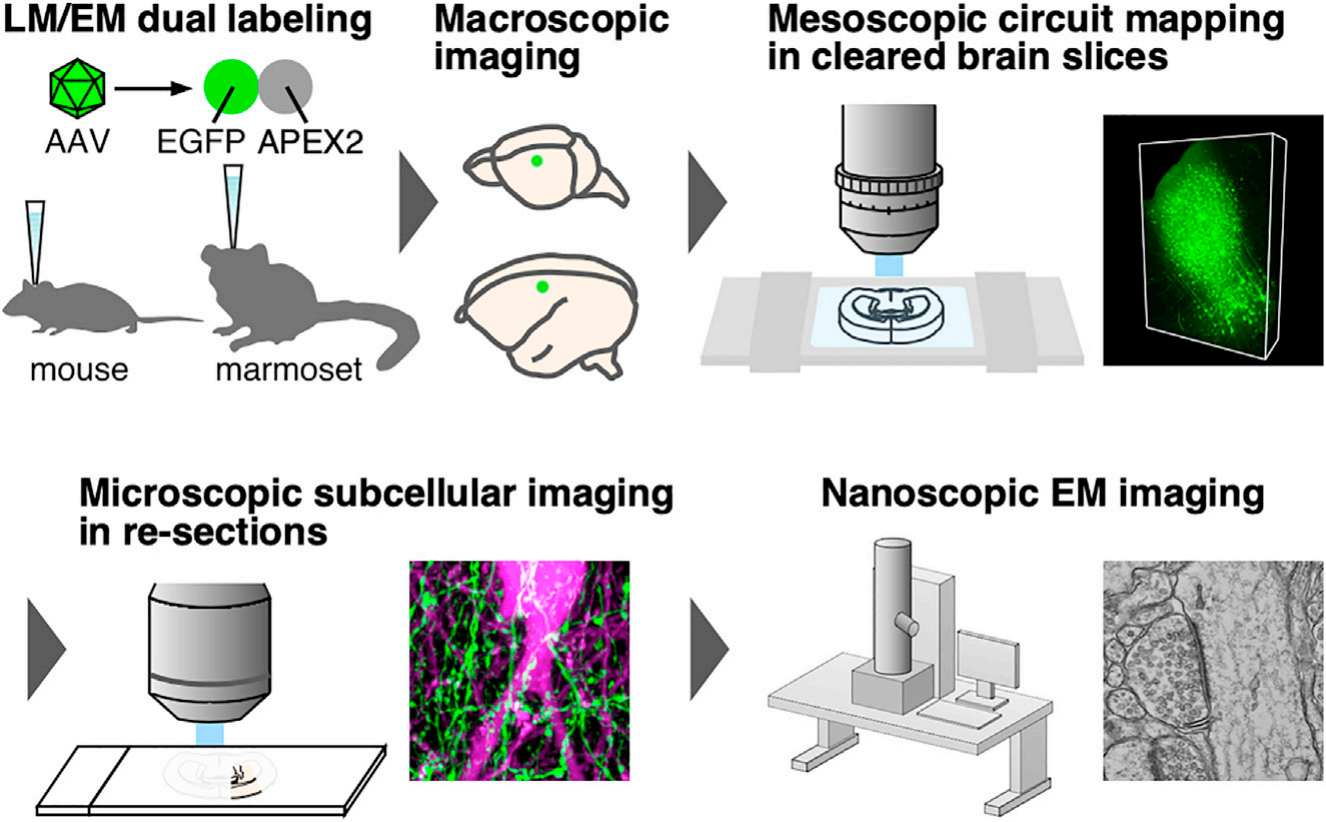
Multi-scale LM/EM neuronal imaging pipeline Overview of multi-scale LM/EM neuronal imaging. Multi-scale neuronal imaging incorporates (1) macroscopic observation, (2) mesoscopic circuit mapping in cleared brain slices, (3) microscopic subcellular imaging in re-sections, and (4) nanoscopic EM imaging. Mouse and marmoset neurons are labeled with an AAV vector carrying a fluorescent and electron-dense genetically encoded CLEM probe, EGFP-APEX2.

To decipher synaptic connectivity spanning mammalian brains across multiple spatial scales, we built a multi-scale LM/EM neuronal imaging technique by coupling successive LM/EM imaging to a tissue clearing technique. Our multi-scale neuronal imaging consists of four main components: (1) macroscopic brain-wide observation, (2) mesoscopic circuit mapping in optically cleared brain slices, (3) microscopic subcellular imaging in re-sections, and (4) nanoscopic EM imaging, allowing seamless integration of structural information from the entire brain to individual synapses (Figure 1). For this, we developed a tissue clearing method that exerts potent clearing capability as well as ultrastructure preservation. We label neurons in mouse and marmoset brains with an adeno-associated virus (AAV) vector carrying a fluorescent and electron-dense genetically encoded correlative light and electron microscopy (CLEM) probe, EGFP-APEX2, for the successive LM/EM imaging. LM/EM dual labeling that remains stable during a clearing procedure is implemented to our imaging pipeline.

### Sca*l*eSF is a tissue clearing method for multi-scale LM/EM neuronal imaging

**Figure 2:**
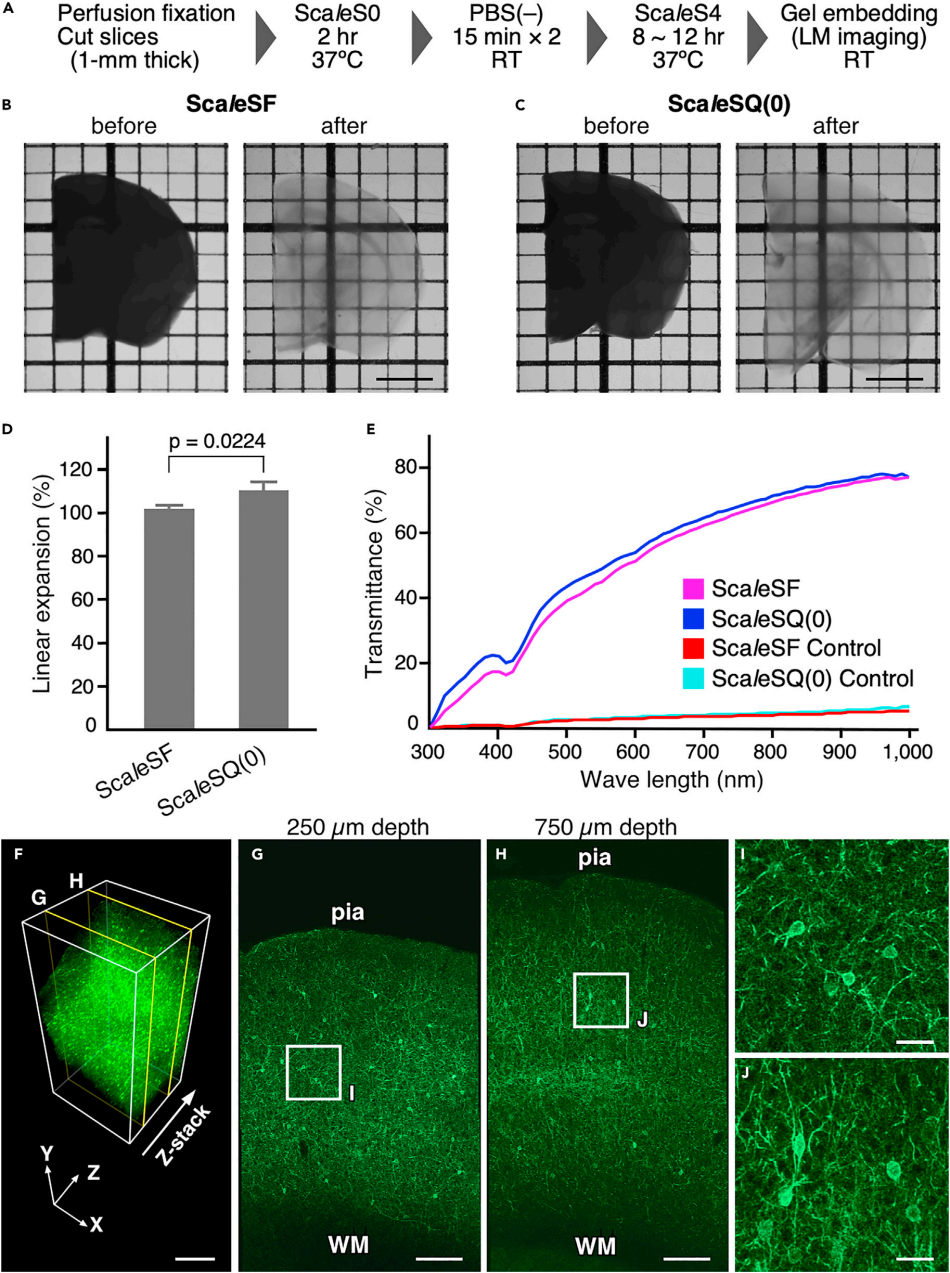
Sca*l*eSF is an isometric and rapid optical clearing method (A) The schedule for tissue clearing with Sca*l*eSF. (B and C) Transmission images of 1-mm-thick brain slices before (left) and after (right) treatment with Sca*l*eSF (B) and Sca*l*eSQ(0) (C). The grid interval is 1 mm. Scale bars, 2 mm. (D) Changes in the size of brain slices after Sca*l*eSF and Sca*l*eSQ(0) treatment (n = 3, Sca*l*eSF; n = 4, Sca*l*eSQ(0); *t* = 3.261, *df* = 5, *P* = 0.0224, two-tailed unpaired Student's t test). Data are represented as means ± SDs. (E) Transmission curves of the PBS-stored control, Sca*l*eSF-, and Sca*l*eSQ(0)-treated mouse brain slices (n = 3 brain hemispheres each). Data are represented as means. (F) Three-dimensional volume rendering of the cerebral cortex of a PV-FGL mouse cleared with Sca*l*eSF. In the PV-FGL mouse, somatodendritic membrane-targeted EGFP expression is driven by a parvalbumin promoter. Scale bar, 500 μm. (G and H) *xy* images in (F) at the depths of 250 μm (G) and 750 μm (H). pia: pia mater, WM: white matter. Scale bars, 200 μm. (I and J) Enlarged views of rectangles in (G) and (H). Scale bars, 40 μm. See also Figures S1–S3.

Multi-scale LM/EM neuronal imaging demands a technique for tissue clearing that achieves a high-level of preservation of ultrastructure and fluorescence signals while simultaneously maintaining potent clearing capability (clearing-preservation spectrum). Of proliferating tissue clearing techniques, an aqueous tissue clearing method, Sca*l*eS, occupies a distinctive position with its effective clearing-preservation spectrum (Hama et al., 2015). However, the clearing protocol of Sca*l*eS, sequential 12-hr incubations in six solutions at 37°C, might lead to less-than-optimal preservation of ultrastructure. Although Sca*l*eSQ(0) is formulated for rapid clearance of brain slices without lipid-extracting detergents, a considerable expansion in sample volume is observed after clearing treatment (Hama et al., 2015), potentially resulting in morphological artifacts. With the goal of minimizing processing time and changes in sample volume, we developed Sca*l*eSF as an isometric and rapid clearing protocol by modifying the clearing procedure of Sca*l*eS (Figure 2A).

The clearing protocol of Sca*l*eSF requires sequential incubations of brain slices in three solutions, Sca*l*eS0 solution, phosphate buffer saline (PBS), and Sca*l*eS4 solution, for a total of 10.5–14.5 hr. Cleared brain slices were embedded in agarose gel dissolved in Sca*l*eS4D25(0) solution (Sca*l*eS4 gel) (Miyawaki et al., 2016). Sca*l*eSF treatment rendered 1-mm-thick mouse brain slices transparent with a similar degree of transparency as that yielded with Sca*l*eSQ(0) (Figures 2B and 2C). Although a modest expansion in sample sizes was observed after Sca*l*eSQ(0) treatment (linear expansion: 110.7 ± 4.1%) (Figures 2C and 2D), the final sizes of brain slices cleared with Sca*l*eSF were approximately 100% of those of the original (linear expansion: 102.5 ± 1.3%) (Figures 2B and 2D) after transient shrinkage and expansion (Figure S1A). The transmission curves of 1-mm-thick mouse brain slices showed that Sca*l*eSF cleared brain slices in a manner comparable with Sca*l*eSQ(0) (Figure 2E). Tissue clearing efficiency of Sca*l*eSF appeared to vary between gray matter and white matter regions of the brain (Figure 2B). Indeed, we found that the white matter region had poor optical transparency than the gray matter region of the cerebral cortex in brain slices cleared with Sca*l*eSF (Figure S2). Although tissues cleared with the original Sca*l*eS protocol can be stably stored in Sca*l*eS4 solution (Hama et al., 2015), brain slices cleared with Sca*l*eSF gradually expanded during storage in the solution (Figure S1B). This expansion could be controlled by embedding the slices in Sca*l*eS4 gel while still maintaining transparency of the cleared slices (Figures S1B and S1C). Thus, Sca*l*eSF is an isometric tissue clearing method with clearing capability comparable with that of Sca*l*eSQ(0).

The fluorescence preservation and clearing capability of Sca*l*eSF were assessed with brain slices of transgenic mice expressing somatodendritic-membrane-targeted enhanced green fluorescent protein (EGFP) in PV-positive neurons (PV-FGL mice) (Kameda et al., 2012). Three-dimensional image acquisition of 1-mm-thick slices collected from the cerebral cortex of the mice was performed using confocal laser scanning microscopy (CLSM) (Figures 2F–2J). The cleared brain slices were placed in a customizable 3D-printed chamber (Figure S3) with Sca*l*eS4 gel. The high resolution of three-dimensional images was demonstrated by *xy* images obtained at different depths (Figures 2G–2J): EGFP targeting of the somatodendritic plasma membrane was discernable even at depths of 250 μm and 750 μm (Figures 2I and 2J), indicating the preservation of both fluorescence signals and membrane structures as well as potent clearing capability of Sca*l*eSF.

**Figure 3:**
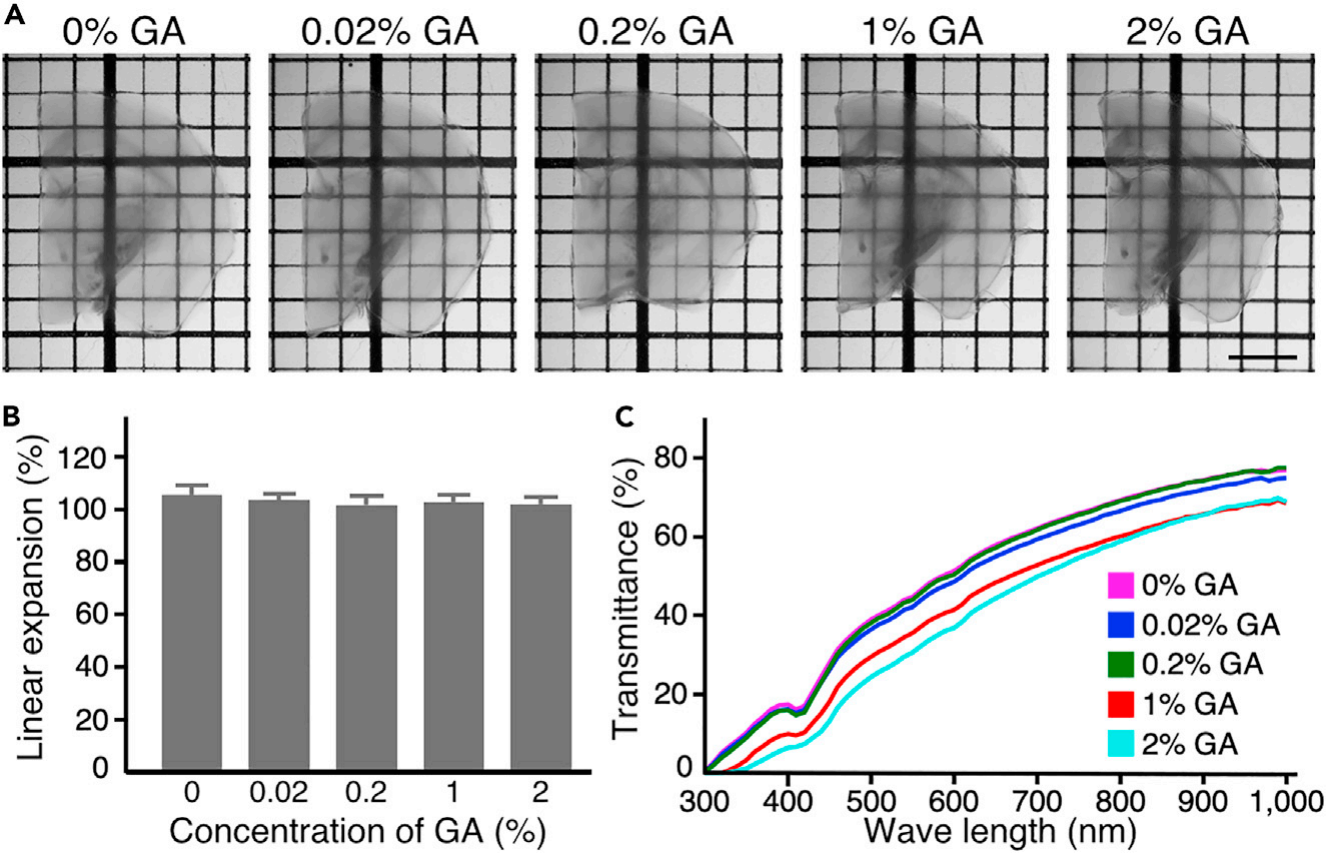
Sca*l*eSF clears brain slices fixed with GA (A) Transmission images of Sca*l*eSF-treated mouse brain slices fixed with 4% PFA or 4% PFA containing GA (0.02%, 0.2%, 1%, or 2%). The thickness of brain slices and the grid interval are 1 mm. Scale bar, 2 mm. (B) Changes in the size of brain slices after Sca*l*eSF treatment (n = 8, GA 0%; n = 8, GA 0.02%; n = 8, GA 0.2%; n = 8, GA 1%; n = 7, GA 2%; n = 4 mice for each condition; *F*_*4,34*_ = 1.975, *P* = 0.121, one-way ANOVA). Data are represented as means ± SDs. (C) Transmission curves of Sca*l*eSF-treated mouse brain slices fixed with 4% PFA or 4% PFA containing GA (0.02, 0.2, 1, or 2%) (n = 3 brain hemispheres each). Data are represented as means.

**Figure 4:**
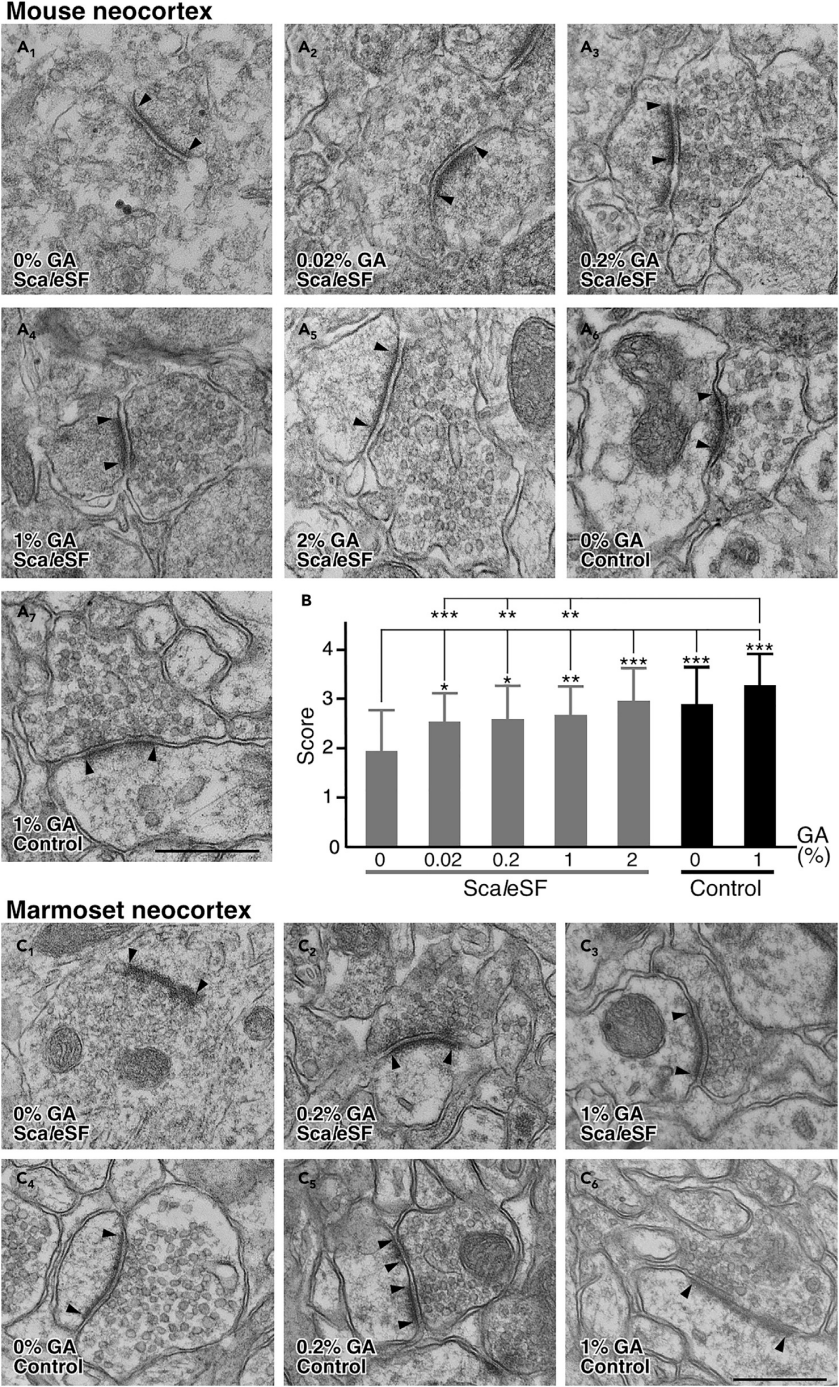
GA improves ultrastructure in both mouse and marmoset brain slices cleared with Sca*l*eSF (A) TEM images of mouse cerebral cortex cleared with Sca*l*eSF (A_1_–A_5_) or stored in PBS(−) (A_6_, A_7_). Mouse brains were fixed with 4% PFA (A_1_, A_6_) or 4% PFA containing GA (0.02%, A_2_; 0.2%, A_3_; 1%, A_4_, A_7_; 2%, A_5_). Arrowheads indicate postsynaptic membranes. Brain slices were cleared with Sca*l*eSF and embedded in Sca*l*eS4 gel for 24 hr, or stored in PBS(−) at 4°C in the analysis. Scale bar, 500 nm. (B) Scoring of membrane continuity of presynaptic terminals for each condition in (A). Over 90%, 50%–90%, 10%–50%, and less than 10% membrane continuity of presynaptic terminals are scored as 4, 3, 2, and 1, respectively (n = 31 synapses, GA 0%, Sca*l*eSF; n = 52 synapses, GA 0.02%, Sca*l*eSF; n = 33 synapses, GA 0.2%, Sca*l*eSF; n = 34 synapses, GA 1%, Sca*l*eSF; n = 31 synapses, GA 2%, Sca*l*eSF; n = 32 synapses, GA 0%, Control [PBS-stored slices]; n = 31 synapses, GA 1%, Control [PBS-stored slices]; n = 3 mice for each condition; *H* = 52.44, *df* = 6, *P* = 1.52 × 10^−9^, Kruskal–Wallis test; ∗*P* < 0.05, ∗∗*P* < 0.01, ∗∗∗*P* < 0.001; Steel–Dwass *post hoc* test). Data are represented as means ± SDs. (C) TEM images of the cerebral cortex of marmosets. Ultrathin sections were prepared from brain slices cleared with Sca*l*eSF (C_1_–C_3_) or stored in PBS(−) (C_4_–C_6_). Marmoset brains were fixed with 4% PFA (C_1_ and C_4_), 4% PFA containing 0.2% (C_2_ and C_5_), or 1% GA (C_3_ and C_6_) (n = 4 marmosets). Brain slices were cleared with Sca*l*eSF and embedded in Sca*l*eS4 gel for 24 hr, or stored in PBS(−) at 4°C in the analysis. Arrowheads indicate postsynaptic membranes. Scale bar, 500 nm. See also Figures S4 and S5.

Fixatives containing GA improve the preservation of ultrastructural morphology (Karnovsky, 1965). However, how GA affects tissue clearing performance and ultrastructural preservation in optically cleared tissues remains unclear. To examine these aspects, first, we tested effects of GA on the clearing capability and isometricity of Sca*l*eSF. Sca*l*eSF treatment rendered GA-fixed brain slices transparent without shrinkage or expansion of their final sizes, albeit less efficiently transparent in brain slices fixed with high concentrations of GA (1 and 2%) (Figure 3). Then, we examined effects of GA on ultrastructural preservation in brain slices cleared with Sca*l*eSF (Figure 4). To this end, Sca*l*eSF-treated mouse brain slices that had been fixed with GA were restored by washing with PBS (deSca*l*ing) (Hama et al., 2011), and synaptic ultrastructure in the cerebral cortex was imaged by TEM. GA improved ultrastructural preservation even in mouse brain slices cleared with Sca*l*eSF (Figure 4A). Raising the concentration of GA in fixatives increased the membrane integrity of presynaptic and postsynaptic structures in the cleared slices (Figures 4A_1_–4A_5_). Scoring the ultrastructural preservation by the membrane continuity of presynaptic terminals demonstrated that, at its low concentration (0.02%), GA improved ultrastructural preservation in the cleared slices to an extent comparable with that in the PBS-stored control slices fixed with paraformaldehyde (PFA) (Figure 4B). We also noticed that the clearing protocol of Sca*l*eSF failed to fully preserve synaptic ultrastructure (Figures 4A_4_, 4A_7_, and 4B). The GA-mediated ultrastructural preservation was more dramatic in brain slices obtained from marmosets (Figure 4C). Without GA, the membrane integrity of presynaptic and postsynaptic structures was severely damaged after clearing with Sca*l*eSF (Figures 4C_1_ and 4C_4_). In contrast, Sca*l*eSF-treated brain slices fixed with 4% PFA containing 0.2% or 1% GA showed nearly complete contiguous membrane integrity (Figures 4C_2_–4C_6_). We also found that an alternative epoxy resin and a different embedding method were compatible with Sca*l*eSF-treated brain slices (Figure S4).

We previously utilized mouse brain tissues conventionally fixed with PFA and found severe degradation of ultrastructures caused by tissue clearing methods, including 3DISCO, CUBIC, and PACT (Hama et al., 2015). Given that GA improved ultrastructural preservation in Sca*l*eSF-treated brain tissues (Figure 4), one might argue that addition of GA can preserve ultrastructures in brain tissues regardless of clearing techniques. We, therefore, investigated ultrastructural preservation in brain tissues treated with CUBIC (Tainaka et al., 2018), PACT (Yang et al., 2014), Sca*l*eSF, or SeeDB (Ke et al., 2013) that had been perfused with 4% PFA containing 1% GA. CUBIC, Sca*l*eSF, and SeeDB are aqueous tissue clearing methods, and PACT is a hydrogel-based tissue clearing method. We did not test ultrastructural preservation in solvent-based clearing methods, such as 3DISCO, because of their deleterious effects on membrane integrity (Ueda et al., 2020a, 2020b). TEM images of the cerebral cortex treated with Sca*l*eSF and SeeDB showed clear ultrastructures such as plasma membrane, synaptic vesicles, mitochondria, and microtubules (Figures S5A and S5B). In contrast, membrane structures were severely damaged in tissues cleared with CUBIC (Figure S5C) and PACT (Figure S5D), even despite perfusion with a fixative containing GA. Only postsynaptic densities (PSDs) and presynaptic membrane of the active zones were observed in these tissues (Figures S5C and S5D). Scoring the membrane continuity of presynaptic terminals demonstrated that ultrastructural preservation in Sca*l*eSF-treated brains was comparable with brain tissues processed by SeeDB (Figure S5E), and membrane integrity was significantly disrupted in brain tissues cleared with CUBIC and PACT compared with Sca*l*eSF- and SeeDB-treated brain tissues (Figure S5E). These findings indicate that addition of GA, rather clearing reagents and/or procedures, such as the amount of lipid-extracting detergents, processing time, and temperature, largely affect ultrastructural preservation and that clearing protocols of CUBIC and PACT were not suitable for EM imaging (Figures S5C and S5D). Molecular preservation in clearing tissues is important for simultaneous interrogation of molecular and structural information. We then compared protein loss in brain tissues cleared with Sca*l*eSF and SeeDB. SeeDB treatment showed the tendency to solubilize more protein in its clearing reagents than Sca*l*eSF (Figure S5F). Collectively, we concluded that Sca*l*eSF is an isometric, rapid, and ultrastructurally preserved clearing method that permits multi-scale LM/EM neuronal imaging.

### APEX2/BT-GO reaction enables the correlated imaging of a fluorescent protein and an osmiophilic polymer in optically cleared tissues

To achieve efficient successive LM/EM imaging in cleared tissues, we designed a genetically encoded probe for CLEM by fusing EGFP in tandem with an engineered ascorbate peroxidase, APEX2 (EGFP-APEX2) (Lam et al., 2015). APEX2 catalyzes the polymerization and local deposition of 3,3-diaminobenzidine (DAB) in the presence of hydrogen peroxidase, which subsequently recruits electron-dense osmium to produce EM contrast. Importantly, APEX2 retains its peroxidase activity even after fixation with GA (Hirabayashi et al., 2018; Joesch et al., 2016; Lam et al., 2015). We used a single AAV vector Tet-Off platform, AAV-SynTetOff (Sohn et al., 2017), for high-level and neuronal expression of the CLEM probe (AAV2/1-SynTetOff-EGFP-APEX2) (Figure S6A). The AAV-SynTetOff platform efficiently visualizes neuronal processes compared with an AAV vector expressing reporter genes under a synapsin-I promoter (Sohn et al., 2017). We tested the feasibility of the vector by stereotactic injections into the mouse primary sensory cortex (S1). Seven to ten days after the injections, 1-mm-thick slices were prepared from the mouse brains perfused with 4% PFA containing 0.2% GA and cleared with Sca*l*eSF. Tissue sections were cut perpendicularly to the deSca*l*ed slices (re-sectioning) and developed in the DAB-Ni^2+^ solution (Figure S6B). Unexpectedly, DAB-Ni^2+^ labeling by APEX2 was much less sensitive than EGFP fluorescence-based detection in Sca*l*eSF-treated re-sections, hampering the correlated fluorescent and bright-field imaging (Figure S6C). We reasoned that clearing with Sca*l*eSF likely accounts for the lower sensitivity of APEX2 because DAB-Ni^2+^ labeling with APEX2 correlated well with EGFP fluorescence in untreated re-sections (Figure S6D). To resolve this problem, we designed an experimental procedure in which biotin molecules are deposited with tyramide signal amplification (TSA) reaction using peroxidase activity of APEX2 (APEX2/BT-GO reaction) prior to Sca*l*eSF treatment, and then re-sections prepared from the cleared slices are processed for ABC/DAB-Ni^2+^ visualization (Figure S6E). APEX2/BT-GO reaction gave remarkably strong DAB-Ni^2+^ labeling even after Sca*l*eSF treatment (Figure S6F). DAB-Ni^2+^ labeling with APEX2/BT-GO reaction was comparable with, or even more sensitive than, EGFP fluorescence in Sca*l*eSF-treated re-sections (compare Figure S6F_1_ with S6F_2_). We further observed DAB-Ni^2+^ labeling in fine subcellular structures such as axons, dendrites, and dendritic spines (Figures S6F_3_ and S6F_4_). Thus, APEX2/BT-GO reaction combined with high-level gene expression by the AAV-SynTetOff platform permits correlated imaging of a fluorescent protein and an osmiophilic polymer in brain slices cleared with Sca*l*eSF.

### Multi-scale LM/EM neuronal imaging in rodent and primate brains

By combining the aforementioned techniques, we implemented the multi-scale LM/EM neuronal imaging of three brain-wide circuits of two different species: mouse striatofugal, mouse callosal, and marmoset corticostriatal projection systems. For accurately tracking targeted structures across multiple spatial scales, we utilized GFP and RFP fluorescence, DAB-Ni^2+^ labeling, and/or endogenous landmarks such as shape of brain structure, blood vessels, and cellular nuclei. Unambiguous correlation of LM and EM datasets was achieved by LM/EM dual labeling with the CLEM probe, EGFP-APEX2.

**Figure 5:**
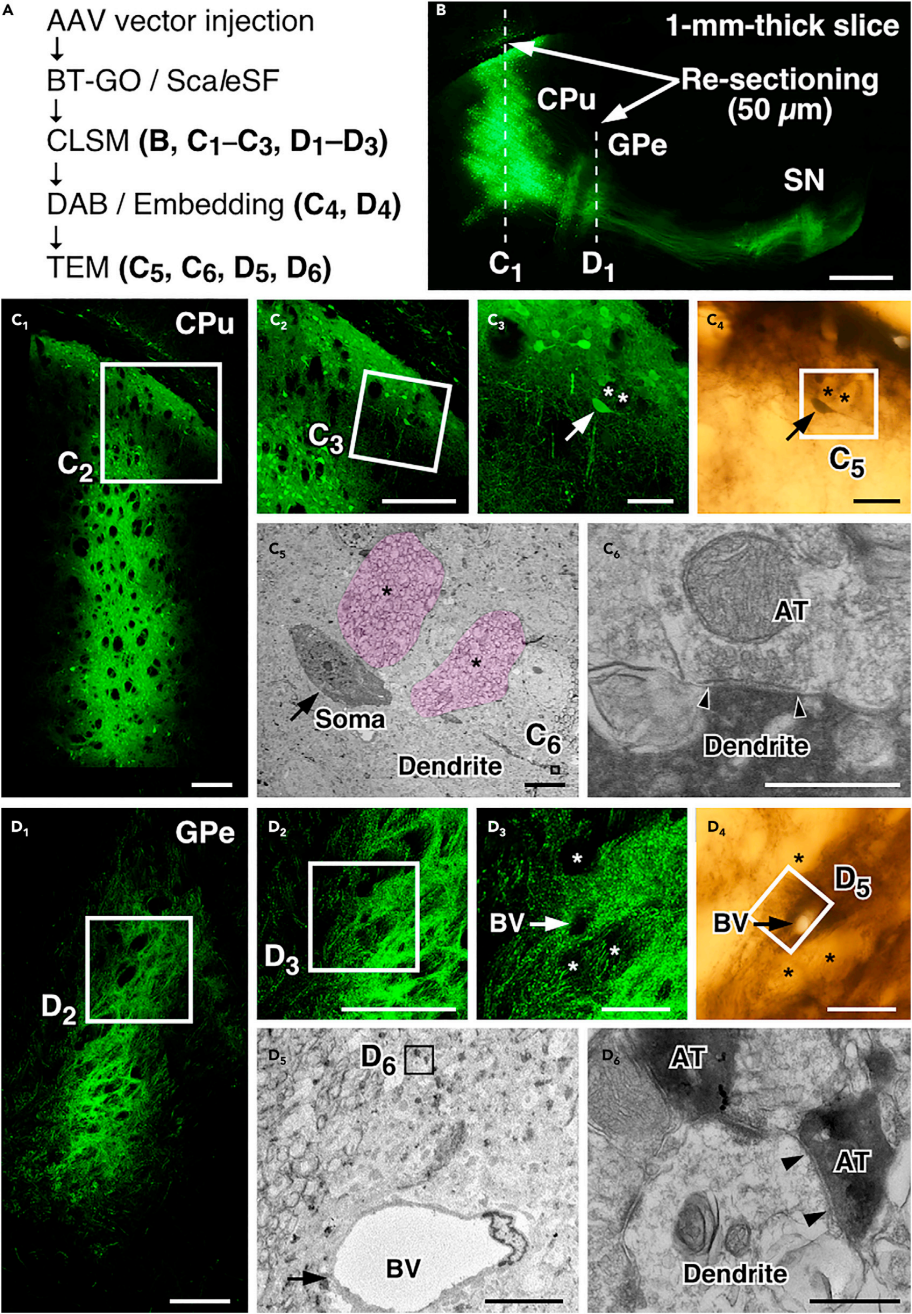
Multi-scale LM/EM neuronal imaging of mouse CPu neurons (A) The procedure of multi-scale LM/EM neuronal imaging of mouse CPu neurons. Brains are fixed with 4% PFA containing 0.2% GA. (B) A maximum intensity projection image of a 1-mm-thick parasagittal brain slice cleared with Sca*l*eSF. The AAV2/1-SynTetOff-EGFP-APEX2 vector is injected into the CPu. Sections of 50 μm thickness are cut along dotted lines. Scale bar, 500 μm. (C and D) Correlated fluorescence (C_1_–C_3_ and D_1_–D_3_), bright-field (C_4_ and D_4_), and TEM images (C_5_, C_6_, D_5_, and D_6_) at the level of CPu (C) and GPe (D). AT: axon terminal, BV: blood vessel. (C_1_ and D_1_) CLSM imaging. Scale bars, 200 μm. (C_2_, C_3_, D_2_, and D_3_) Enlarged views of rectangles in (C_1_), (C_2_), (D_1_), and (D_2_), respectively. Scale bars, 200 μm in (C_2_ and D_2_) and 50 μm in (C_3_ and D_3_). (C_4_ and D_4_) DAB-Ni^2+^ labeling with APEX2/BT-GO reaction. Scale bars, 50 μm. (C_5_ and D_5_) A TEM image of the rectangle in (C_4_) and (D_4_). Scale bars, 10 μm. (C_6_ and D_6_) A high magnification image of the rectangle in (C_5_) and (D_5_). A neuron indicated by arrows in (C_3_ and C_4_) is targeted. Asterisks in (C_3_–C_5_) and (D_3_ and D_4_) indicate the same bundles of axonal fibers in (C) and (D), respectively. Arrows in (D_3_–D_5_) indicate the identical blood vessel. Arrowheads in (C_6_ and D_6_) indicate postsynaptic membranes. Scale bars, 500 nm. See also Figures S6 and S7.

The caudate-putamen (CPu) is the primary input structure of the basal ganglia (Alexander and Crutcher, 1990); it receives glutamatergic afferents from the cerebral cortex and thalamus and sends GABAergic efferents to the external segment of the globus pallidus (GPe), entopeduncular nucleus (EP), and substantia nigra (SN). The striatofugal projection system was thus used as a model to test the present imaging technique. Figure 5A presents the workflows for the multi-scale LM/EM neuronal imaging of murine striatal circuitry. Four weeks after the injections of the AAV2/1-SynTetOff-EGFP-APEX2 vector into the mouse CPu, the brains were fixed with 4% PFA containing 0.2% GA to improve ultrastructural preservation. Parasagittal slices (1-mm thick) were prepared from the brains, and biotin molecules were deposited with APEX2/BT-GO reaction. The slices were cleared with Sca*l*eSF, and then macroscopic and mesoscopic neural circuit mapping was conducted by CLSM (Figure 5B). After perpendicular re-sectioning of the imaged slices (dotted lines in Figure 5B), high-resolution image stacks were collected to document the detailed morphologies of the labeled neurons (Figures 5C_1_–5C_3_ and 5D_1_–5D_3_). The imaged re-sections were processed for ABC/DAB-Ni^2+^ reaction using the deposited biotin molecules by APEX2/BT-GO reaction and embedded in an epoxy resin (Figures 5C_4_ and 5D_4_). Ultrathin sections were prepared from the re-sections and imaged with TEM at a nanometer resolution (Figures 5C_5_, 5C_6_, 5D_5_, and 5D_6_).

We first performed the multi-scale LM/EM neuronal imaging of synaptic inputs to striatal neurons and synaptic outputs to the GPe (Figure 5). CLSM imaging in a Sca*l*eSF-treated brain slice clearly showed the striatofugal projection system: EGFP-labeled fibers arising from the CPu extended caudally to the brainstem, forming dense terminal fields in the GPe and SN (Figure 5B). We targeted a neuron in the dorsal CPu on the input side (Figures 5C_1_–5C_4_) and succeeded in performing EM imaging of the synaptic ultrastructure of the targeted dendrite (Figures 5C_5_ and 5C_6_). The striatopallidal pathway, a GABAergic inhibitory connection between the CPu and GPe, was mapped in the Sca*l*eSF-treated slice (Figure 5B). A re-section from the imaged slice showed varicose axon arborizations (Figures 5D_1_–5D_3_) of the labeled neurons in the GPe. Following ABC/DAB-Ni^2+^ reaction (Figure 5D_4_), axon terminals filled with the dark DAB precipitates were imaged with TEM (Figures 5D_5_ and 5D_6_). We confirmed symmetric synapses, which are characterized by the absence of PSD and the narrow synaptic cleft, on a dendrite of the GPe neuron (Figure 5D_6_). We then performed multi-scale LM/EM neuronal imaging of striatonigral fibers on another cleared slice (Figure S7). Myelin is a protein-lipid bilayer sheath that extends from oligodendrocytes and Schwann cells. Although an immunofluorescence study shows the unmyelinated character of striatonigral fibers (Miyazaki et al., 2014), there is no direct evidence that striatonigral fibers are unmyelinated by EM observation. We thus applied our multi-scale LM/EM neuronal imaging to examine whether striatonigral fibers are indeed unmyelinated. A re-section at the level of medial forebrain bundle (MFB) was prepared from the imaged slice and processed for successive LM/EM imaging (Figures S7B and S7C). Targeting axonal bundles near the optic tract (OT) (Figures S7C_1_ and S7C_2_), we found that almost all the darkly stained axons were unmyelinated (Figures S7C_3_ and S7C_4_). We also applied multi-scale neuronal imaging to GABAergic inhibitory synapses between striatal projection neurons and SN neurons (Figures S7B and S7D). Beginning with the mapping of the striatonigral projection in the cleared slice (Figure S7B), varicose axon arborizations were visualized in a re-section at the level of SN (Figure S7D_1_), and a DAB-labeled axon terminal forming a symmetric synapse with a dendritic process was successively imaged (Figures S7D_2_–S7D_4_).

**Figure 6:**
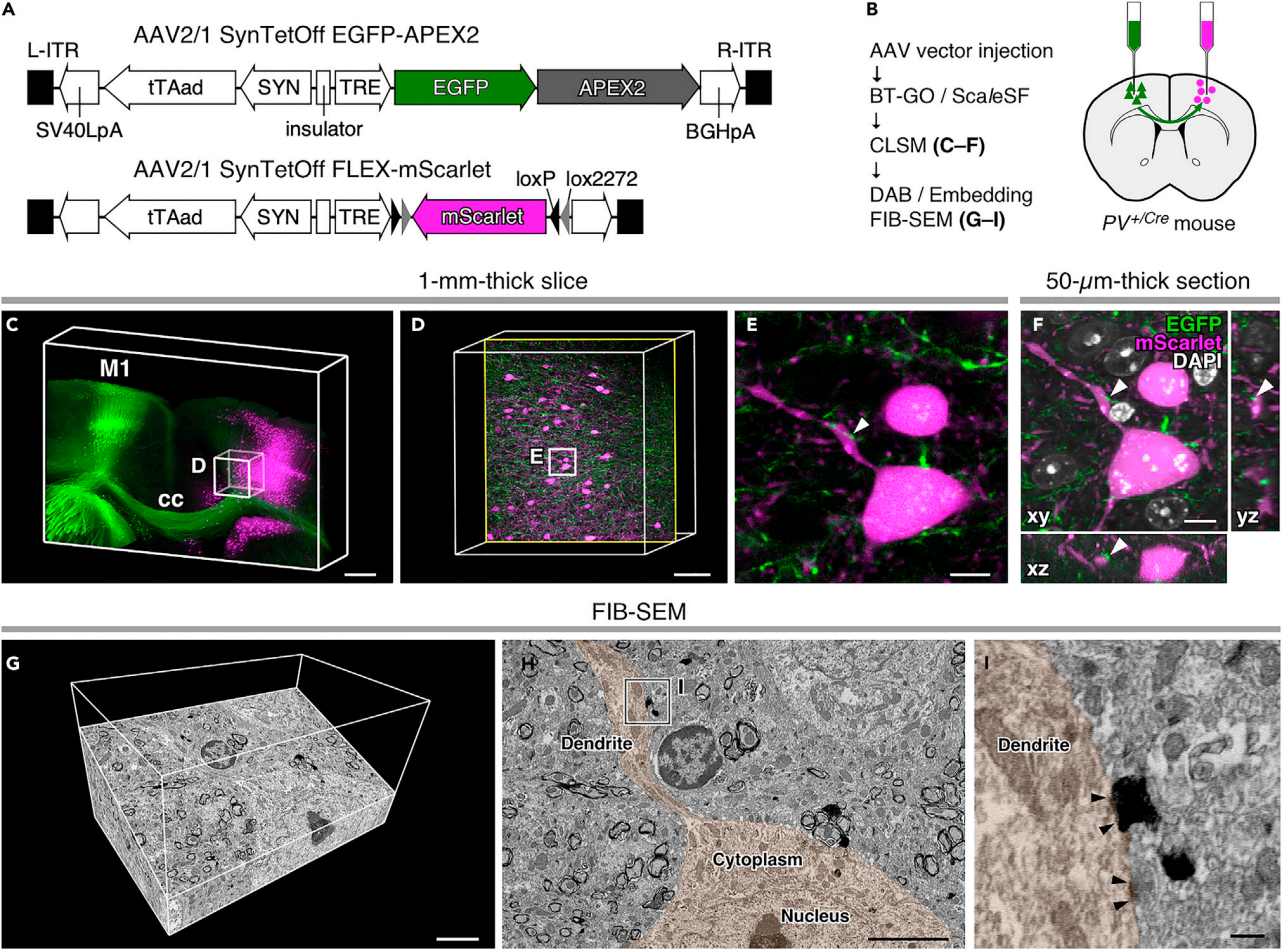
Multi-scale LM/EM neuronal imaging of a mouse callosal synaptic input onto a PV neocortical interneuron (A) The AAV2/1-SynTetOff-EGFP-APEX2 and AAV2/1-SynTetOff-FLEX-mScarlet vectors. BGHpA: polyadenylation signal derived from the bovine growth hormone gene, ITR: inverted terminal repeat, SV40LpA: polyadenylation signal of Simian virus 40 late, SYN: human synapsin I promoter, TRE: tetracycline-responsive element, tTAad: an improved version of a tetracycline-controlled transactivator. (B) The procedure of multi-scale LM/EM neuronal imaging of mouse callosal synaptic inputs onto PV neocortical interneurons. Brains are fixed with 4% PFA containing 0.2% GA. (C–E) CLSM imaging of a 1-mm-thick brain slice. (C) Three-dimensional volume rendering of the M1 of a *PV*^*+/cre*^ mouse injected with the AAV vectors. cc: corpus callosum. Scale bar, 500 μm. (D) An enlarged and high-resolution image of the box in (C). The image is rotated 90° in a counterclockwise direction with respect to (C). Scale bar, 100 μm. (E) A higher magnification image of the rectangle in (D). An optical section with an axodendritic apposition is shown. Scale bar, 10 μm. (F) CLSM imaging in a re-section. A 50-μm-thick section was cut parallel to the *xy* plane from the slice imaged in (C–E). Orthogonal views of the *xz* (bottom) and *yz* (right) planes are also shown. White arrowheads in (E and F) indicate the same axodendritic apposition. Scale bar, 10 μm. (G–I) FIB-SEM tomography of the axodendritic apposition. (G) A three-dimensional volume rendering image. Scale bar, 5 μm. (H) An oblique-slice view. Scale bar, 5 μm. (I) An enlarged view of the rectangle in (H). Arrowheads in (I) indicate the PSD. The profiles of postsynaptic dendrite and soma with the targeted axodendritic apposition (white arrowheads in E and F) are pseudocolored in (H and I) for clarity. Black arrowheads indicate postsynaptic membranes. Scale bar, 500 nm. See also Video S1.

Our multi-scale LM/EM imaging allows for LM imaging of substantial tissue volume at high-resolution and subsequent EM observation of targeted structures, facilitating the capture of scarce structures with nanoscale resolution. Callosal projection neurons are a heterogeneous population of neocortical projection neurons that interconnect the two hemispheres of the cerebral cortex (Innocenti, 1986). Notably, callosal inputs onto GABAergic neocortical interneurons are scant: the vast majority of callosal terminals establish synapses onto dendritic spines, likely those of excitatory pyramidal neurons, whereas the remainder synapses onto dendritic shafts of spiny and aspiny neurons in mice (Czeiger and White, 1993; White and Czeiger, 1991). We, therefore, chose callosal synaptic inputs onto a GABAergic neocortical interneuron subtype, PV neocortical interneurons, in mice as scarce structures with nanoscale resolution and tracked them in a targeted way across multiple spatial scales by the successive LM/EM imaging (Figure 6). The AAV2/1-SynTetOff-EGFP-APEX2 vector was injected into the primary motor cortex (M1), and the AAV2/1-SynTetOff-FLEX-mScarlet was injected into the contralateral M1 of *PV*^*+/Cre*^ mice to label callosal axons with EGFP and PV neocortical interneurons with mScarlet (Figures 6A and 6B). The mouse brains were fixed with 4% PFA containing 0.2% GA. CLSM imaging in Sca*l*eSF-treated brain slices mapped the callosal projection system: EGFP-labeled axons arising from the M1 passed through the corpus callosum and projected to the homotopic contralateral cortex, where mScarlet-labeled PV interneurons were located (Figures 6C and 6D). We screened numerous (>3000) serial *xy* images (121 × 121 μm^2^) at different *z* positions in a 1-mm-thick brain slice cleared with Sca*l*eSF and identified an apposition between a callosal axon terminal and a dendrite of PV neocortical interneuron (Figure 6E, arrowhead). After re-sectioning the imaged slices parallel to the *xy* plane (parallel re-sectioning) followed by counterstaining with 4′,6-diamidino-2-phenylindole (DAPI), the possible synaptic contact was validated with high-resolution imaging with CLSM (Figure 6F, arrowheads). Following ABC/DAB-Ni^2+^ reaction and resin embedding, the re-section was subjected to FIB-SEM imaging (Figures 6G–6I and Video S1). The CLSM image in the slice exactly matched the FIB-SEM tomogram (compare Figures 6E with 6H): mScarlet fluorescence corresponded to the SEM profile of membrane structure, and EGFP fluorescence correlated well with the DAB-Ni^2+^ precipitates. Correlation of CLSM in the Sca*l*eSF-treated brain slice, CLSM in the re-section, and FIB-SEM datasets demonstrated the preservation of structural integrity throughout successive LM/EM imaging (Figures 6C–6I). The axodendritic apposition between the callosal axon and the PV neocortical interneuron (Figures 6E and 6F, arrowheads) actually formed a synaptic contact: we observed an asymmetric synaptic specialization, which is characterized by the presence of PSD, at the apposition between the axon terminal filled with electron-dense DAB precipitates and the dendrite in an FIB-SEM tomogram (Figure 6I).

Video S1. Three-dimensional data of ultrastructures shown in Figure 6G, related to Figure 6 This movie demonstrates an image stack of the targeted ultrastructures, obtained by FIB-SEM. Voxel size: 10 nm/voxel

**Figure 7:**
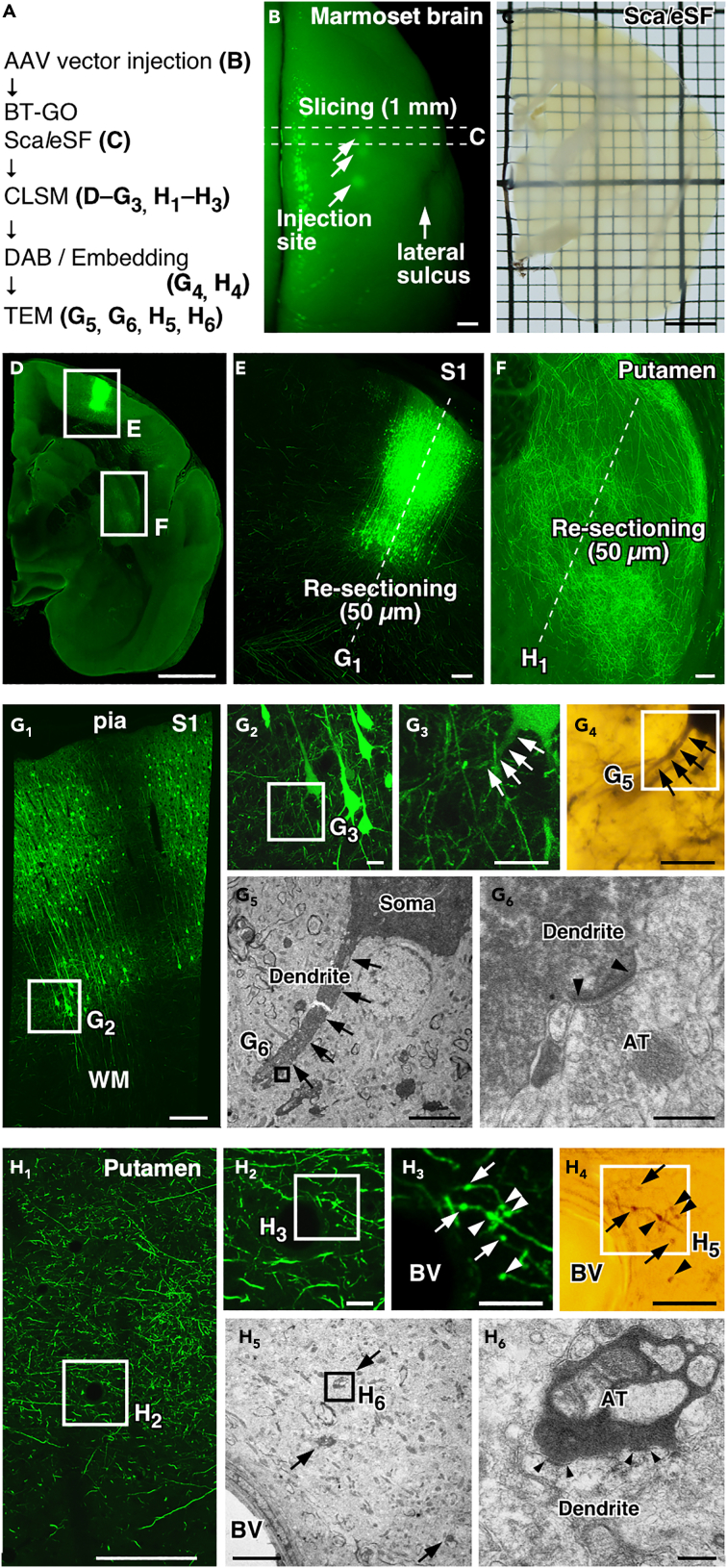
Multi-scale LM/EM neuronal imaging of cortical neurons in a marmoset brain (A) The procedure of multi-scale LM/EM neuronal imaging. Brains are fixed with 4% PFA containing 0.2% GA. (B) EGFP fluorescence (arrowheads) in a marmoset brain 6 weeks after injections of the AAV2/1-SynTetOff-EGFP-APEX2 vector. A 1-mm-thick brain slice is cut along dotted lines. Scale bar, 3 mm. (C) A transmission image of the slice cleared with Sca*l*eSF. Scale bar, 3 mm. (D) A maximum intensity projection image of the cleared slice. Scale bar, 3 mm. (E and F) Enlarged views of the S1 (E) and putamen (F). Sections of 50 μm thickness are cut along dotted lines. Scale bars, 200 μm. (G and H) Correlated fluorescence (G_1_–G_3_ and H_1_–H_3_), bright-field (G_4_ and H_4_), and TEM images (G_5_, G_6_, H_5_, and H_6_) in the S1 (G) and putamen (H). AT: axon terminal, BV: blood vessel. (G_1_ and H_1_) CLSM imaging. Scale bars, 200 μm. (G_2_, G_3_, H_2_, and H_3_) Enlarged views of the rectangles in (G_1_), (G_2_), (H_1_), and (H_2_), respectively. Scale bars, 20 μm. (G_4_ and H_4_) DAB-Ni^2+^ labeling with APEX2/BT-GO reaction. Scale bars, 20 μm. (G_5_ and H_5_) TEM images of the rectangle in (G_4_) and (H_4_). Scale bars, 5 μm. (G_6_ and H_6_) A high magnification image of the rectangle in (G_5_) and (H_5_). A synaptic structure (arrowheads in G_6_) in a dendrite (arrows in G_3_–G_5_) of a pyramidal neuron is targeted in (G), and synaptic structures (arrowheads in H_6_) between a cortical axon and a putamen dendrite are targeted in (H). Arrows in (H_3_–H_5_) and arrowheads in (H_3_ and H_4_) indicate the same presynaptic terminals. Scale bars, 300 nm.

Multi-scale LM/EM neuronal imaging should be effective in large-brained animals such as primates. The marmoset is becoming increasingly popular as a model organism in neuroscience research fields because of its social cognitive abilities and amenability to genetic manipulation (Miller et al., 2016; Okano et al., 2016). We finally demonstrated the applicability of our multi-scale neuronal imaging in marmoset brains (Figure 7). The AAV2/1-SynTetOff-EGFP-APEX2 vector was injected into multiple neocortical sites of marmosets, and the brains were fixed with 4% PFA containing 0.2% GA after 6 weeks of the injections. We identified clusters of neuronal elements visualized by EGFP-APEX2 expression in a macroscopic whole-brain image (Figure 7B). The brains were then cut into 1-mm-thick coronal slices, and those containing injection sites were cleared with Sca*l*eSF (Figure 7C). Neural circuit mapping with CLSM revealed the corticostriatal projection in the cleared slice: EGFP-labeled axons arising from the S1 extended subcortically and formed a dense terminal field in the putamen (Figure 7D–7F). After deSca*l*ing with PBS, the imaged slice was cut into sections for subcellular imaging with CLSM (Figures 7G_1_ and 7H_1_). High-resolution image stacks in the re-sections documented the detailed morphologies of labeled neurons: pyramidal-shaped somata, apical and basal dendrites emanating from somata, and axonal projections extending basally and horizontally in the S1 (Figures 7G_1_–7G_3_), and axon terminal arborizations and axonal boutons in the putamen (Figures 7H_1_–7H_3_). Of these structures, we targeted a basal dendrite of a pyramidal neuron on the input side (arrows in Figure 7G_3_) and a corticostriatal axonal bouton on the output side (arrows in Figure 7H_3_) for subsequent EM imaging. Following ABC/DAB-Ni^2+^ reaction and resin embedding (Figure 7G_4_ and 7H_4_), ultrathin sections were prepared from the re-sections and further processed for EM (Figure 7G_5_, 7G_6_, 7H_5_, and 7H_6_). We observed an asymmetric synapse, which typically mediates glutamatergic neurotransmission, on the targeted dendrite filled with electron-dense DAB precipitates (Figure 7G_6_), as well as an asymmetric synapse between a corticostriatal axon terminal and a striatal dendrite (Figure 7H_6_). Given macroscopic imaging of centimeter-sized marmoset brains (3 cm length and 2 cm width; Figure 7B) and TEM imaging of synapses with nanometer resolution (1.2 nm/pixel; Figure 7G_6_ and 7H_6_), we succeeded in multi-scale LM/EM neuronal imaging over seven orders of magnitude.

## Discussion

The imaging scale required for deciphering brain-wide synaptic connectivity in the mammalian brain exceeds several orders of magnitude (Lichtman and Denk, 2011). We overcame the technical challenges associated with this requirement by coupling a tissue clearing method with successive LM/EM imaging. Our multi-scale LM/EM neuronal imaging enables brain-wide connectomic analysis by simultaneous interrogation of their neural circuit structures with LM and synaptic connectivity with EM. The feasibility of the multi-scale neuronal imaging was demonstrated in the mouse striatofugal projection system. Beginning with mapping the projection in cleared brain tissues, we anterogradely imaged the detailed morphologies of labeled neurons with CLSM and targeted nanoscopic structures such as synapses and myelin sheaths (Figures 5 and S7). As demonstrated by the application to marmoset brains (Figure 7), our multi-scale imaging should be effective in connectomic analysis of large-brained animals. Our multi-scale LM/EM imaging that is featured with LM imaging of substantial tissue volume at high-resolution followed by subsequent EM imaging at a nanometer resolution allowed us to capture scarce synaptic contacts and callosal synaptic inputs onto PV neocortical interneurons in mice (Figure 6). Our multi-scale LM/EM imaging can complement current comprehensive connectomic analysis (Kornfeld and Denk, 2018; Kubota et al., 2018). Although current comprehensive approaches with EM alone are mainly applied to small pieces of brain tissues (Helmstaedter et al., 2013; Kasthuri et al., 2015; Motta et al., 2019; Schmidt et al., 2017), the present imaging modality makes it possible to describe synaptic connectivity of brain-wide circuits by integrating seamlessly structural information with different spatial scales in a reasonable amount of time without specialized equipment.

Sca*l*eSF, a rapid, isometric, and ultrastructurally preserved clearing technique, facilitated multi-scale LM/EM neuronal imaging. Multi-scale LM/EM imaging requires a tissue clearing method that allows for the preservation of ultrastructure and fluorescence signals. However, most tissue clearing methods, especially protocols featuring high clearing capabilities, aggressively remove lipids and pigments for extensive tissue clarification (Ueda et al., 2020a, 2020b), compromising ultrastructural preservation (Hama et al., 2015; Neckel et al., 2016) (but see ref [Chung et al., 2013]) and this ultrastructural degradation was not rescued by perfusion with fixative containing GA (Figures S5A– S5E). Compared with solvent- and hydrogel-based tissue clearing methods, aqueous tissue clearing methods surpass in preserving fluorescence signals and tissue integrity (Ueda et al., 2020a, 2020b). Although aqueous tissue clearing methods containing minimal lipid-extracting detergents have been reported (Ueda et al., 2020a, 2020b), Sca*l*eSF is the most suitable for use with effective and efficient multi-scale LM/EM imaging, i.e., isometricity, resistance against GA, ultrastructure and molecular preservation, fast processing, and a clearing capability for 1-mm-thick brain slices. The 1-mm thickness of brain slices used in this study is satisfactory enough to recover all of dendritic arbors and inhibitory interneuron axonal arbors of the rodent and carnivore cerebral cortex in their entirely (Mihaljevic et al., 2019; Stepanyants et al., 2009), providing rich structural information on neural circuit architecture. Sca*l*eSF cleared brain slices fixed with various concentrations of GA (Figure 3). The concentration of GA in fixatives should be determined for each experiment. Although high-concentration GA fixation provided superior ultrastructure preservation in brain slices cleared with Sca*l*eSF (Figure 4), GA in low concentrations might be optimal for immunoelectron microscopic studies. GA fixation typically increases tissue autofluorescence and complicates imaging of structures with low signals (Park et al., 2019). In addition, clearing efficacy of Sca*l*eSF was decreased in brain slices fixed with high concentrations of GA (1% and 2%) (Figure 3).

Successive LM/EM imaging can be performed efficiently with fluorescent and electron-dense genetically encoded CLEM probes. LM/EM dual labeling with a single protein enables the unambiguous correlation of LM and EM datasets. Although the correlation can be achieved by endogenous and artificial landmarks, these techniques require additional labeling for endogenous landmarks and/or specialized equipment (Bishop et al., 2011; Karreman et al., 2016; Luckner et al., 2018). Genetically encoded CLEM probes for our multi-scale LM/EM neuronal imaging should be stable in cleared samples. APEX2 retains peroxidase activity even upon fixation with GA (Hirabayashi et al., 2018; Joesch et al., 2016; Lam et al., 2015), rendering APEX2 fusion constructs with fluorescent proteins as good candidates for the CLEM probes. However, we found that peroxidase activity of APEX2 was unexpectedly low after clearing with Sca*l*eSF (Figures S6C and S6D). Hence, we introduced APEX2/BT-GO reaction prior to the clearing treatment to deposit biotin molecules with TSA reaction using peroxidase activity of APEX2 (Figure S6E). APEX2/BT-GO reaction provided remarkably strong DAB-Ni^2+^ labeling while maintaining EGFP fluorescence (Figure S6F) that achieved LM/EM dual labeling in brain slices even cleared with Sca*l*eSF.

Simultaneous interrogation of molecular and structural information is required for the advancement of connectomic analysis. However, molecular information is often lost in connectomic analysis with EM alone, and LM lacks nanoscale resolution necessary to identify a single synapse. Our multi-scale LM/EM neuronal imaging overcomes the deficiency of both analyses. Sca*l*e technologies achieve stable tissue preservation for immunohistochemical labeling on re-sections prepared from deSca*l*ed tissues (Hama et al., 2011, 2015) and can thus be used to collect both molecular and structural information. Furthermore, our labeling approach with genetically encoded probes can be applied to a library of Cre driver lines, providing us with a genetic handle on studying neural circuit structure and synaptic connectivity of specific neuronal types. Indeed, we identified and tracked mouse callosal inputs onto a GABAergic neocortical interneuron subtype, PV neocortical interneurons, by injecting a flexed AAV vector coding for mScarlet into *PV*^*+/Cre*^ mouse brains (Figure 6). The high-level preservation of fluorescence signals and ultrastructure in Sca*l*eSF-treated brain slices (Figures 2, 3, 4, and S5) could be amenable to *post hoc* molecular mapping with high accuracy on re-sections, such as array tomography (Micheva and Smith, 2007) and super-resolution imaging (Schermelleh et al., 2010).

In summary, we developed and validated multi-scale LM/EM neuronal imaging for connectomic analysis of neuronal circuits spanning the mammalian brain. Our imaging modality will significantly advance the understanding of brain-wide connectivity by zooming-in on the objects over several orders of magnitude.

### Limitations of study

In the present study, we developed Sca*l*eSF as an isometric and rapid clearing protocol and succeeded in a high-level of preservation of ultrastructure and fluorescence signals (Figures 2, 3, 4, and S5). However, two challenges remain in the clearing protocol. The first is the advanced preservation of ultrastructure: a slight but statistically significant degradation of the ultrastructure in brain slices cleared with Sca*l*eSF (Figure 4B) leaves room for further improvement. The second is the scaling of the clearing protocol: Sca*l*eSF was developed for clearing brain slices, not for a whole brain. Although 1-mm-thick brain slices provide good knowledge of dendritic and local axonal arbors, information about long-range projections is fragmentary and incomplete in the slices (Kuramoto et al., 2009; Lin et al., 2018; Matsuda et al., 2009; Stepanyants et al., 2009; Winnubst et al., 2019). Direct perfusion of clearing reagents that enhances clearing capability (Tainaka et al., 2014; Yang et al., 2014; Zhao et al., 2020) might permit whole-brain clearing accompanied with preserved ultrastructure and fluorescence signals.

Our LM/EM dual labeling coupling a genetically encoded CLEM probe, EGFP-APEX2, with APEX2/BT-GO reaction gave strong EM contrast introduced in the form of osmiophilic polymers throughout the cytoplasm (Figures 5, 6, 7, and S7). Despite its potent LM/EM dual labeling, APEX2 BT-GO reaction itself and permeabilization with a lipid-extracting detergent can potentially damage cellular ultrastructure. Distinct CLEM techniques avoiding osmium fixation and Epon embedding, such as resins that retain fluorophore (Micheva and Smith, 2007), osmium-resistant fluorescent proteins (Fu et al., 2020; Paez-Segala et al., 2015; Tanida et al., 2020a, 2020b), and Tokuyasu cryosectioning (Tokuyasu, 1973), might be useful for improving ultrastructure preservation. Although cytoplasmic labeling with DAB facilitates the identification of targeted structures, the labeling requires detergents and may interfere with visualization of ultrastructural features of synapses such as active zones, synaptic vesicle morphologies, and PSDs. CLEM constructs that express a fluorescent protein throughout the cytoplasm and target to peroxidase to specific subcellular compartments (Zhang et al., 2019) would make it possible simultaneous visualization of neural circuit structures and ultrastructural properties of synaptic arrangements as well as multiplexed labeling in LM/EM.

## STAR★Methods

### Key resources table

**Table:** 

REAGENT or RESOURCE	SOURCE	IDENTIFIER
**Bacterial and virus strains**		

AAV2/1-SynTetOff-EGFP-APEX2	This paper	N/A
AAV2/1-SynTetOff-FLEX-mScarlet	This paper	N/A

**Chemicals, peptides, and recombinant proteins**		

1,4-diazabicyclo[2.2.2]octane (DABCO)	Wako Pure Chemical Industries	049-25712
2,2′-Azobis[2-(2-imidazolin-2-yl)propane] dihydrochloride	Wako Pure Chemical Industries	VA-044
Acrylamide	Bio-Rad	161-0140
Agar	Nacalai Tesque	01028-85
Agarose	TaKaRa Bio	L03
Ampicillin	Meiji Seika pharma	N/A
α-thioglycerol	Sigma-Aldrich	M1753
Atipamezole	Zenoaq	N/A
Atropine	Nipro ES Pharma	N/A
Avidin-biotinylated peroxidase complex (ABC)	Vector	PK-6100
ß-D-glucose	Wako Pure Chemical Industries	049-31165
Biotin-NHS	CALBIOCHEM	203112
Bovine serum albumin	Nacalai Tesque	01863-77
Butorphanol	Meiji Seika Pharma	N/A
CUBIC-L	Tokyo Chemical Industry	T3740
CUBIC-R + (M)	Tokyo Chemical Industry	T3741
DAB·4HCl	Dojindo	347-00904
DAPI	Thermo Fisher Scientific	D1306
Dexamethasone	Aspen Japan	N/A
Diclofenac sodium	Novartic Japan	11147700J1057
Dimethyl sulfoxide	Nacalai Tesque	13407-45
Dulbecco's modified Eagle's medium	Thermo Fisher Scientific	11965-092
Durcupan	Sigma-Aldrich	44610
Fetal bovine serum	Sigma-Aldrich	173012
Fructose	Sigma-Aldrich	F0127
γ-cyclodextrin	Wako Pure Chemical Industries	037-10643
Gentamicin	Nichi-Iko Pharmaceutical	N/A
Glucose oxidase	Nacalai Tesque	16831-14
Glutaraldehyde	Nacalai Tesque	17003-92
Glycerol	Sigma-Aldrich	G9012
Heparin	Mochida Pharmaceutical	224122458
Isoflurane	Pfizer	N/A
Ketamine	Daiichi Sankyo Propharma	D00711
Lactated Ringer's solution	Terumo	N/A
L-glutamine	Thermo Fisher Scientific	25030-081
LR clonase II	Thermo Fisher Scientific	11791020
Luveak-812	Nacalai Tesque	20829-05
Luveak-DDSA	Nacalai Tesque	14423-95
Luveak-DMP-30	Nacalai Tesque	14425-75
Luveak-MNA	Nacalai Tesque	14424-85
Medetomidine	Zenoaq	N/A
MEM Non-Essential amino acid	Thermo Fisher Scientific	11,140-050
Methyl-β-cyclodextrin	Tokyo Chemical Industry	M1356
Midazolam	Astellas Pharm	N/A
Monoethanolamine (2-aminoethanol)	Nacalai Tesque	23405-55
Nickel ammonium sulfate	Nacalai Tesque	24217-82
OptiPrep	Axis-Shield	1114542
Osmium Tetroxide (OsO_4_)	Nacalai Tesque	25746-06
Paraformaldehyde	Merck Millipore	1.04005.1000
PBS(–)	Nacalai Tesque	27575-31
Penicillin-treptomycin	Thermo Fisher Scientific	15070-063
Polyethylenimine	Polysciences	23966
Propylene oxide	Nacalai Tesque	29223-55
Protease inhibitor cocktail	Sigma-Aldrich	P8849
Rigid acrylic resin	Keyence	AR-M2
Sodium azide	Nacalai Tesque	31233-55
Sodium dodecyl sulfate	Nacalai Tesque	31607-65
Sodium pentobarbital	Kyoritsu Seiyaku	N/A
Sorbitol	Nacalai Tesque	06286-55
Triton X-100	Nacalai Tesque	35501-15
TrypLE Express	Thermo Fisher Scientific	12605-010
Tyramine hydrochloride	Sigma Aldrich	T2879-1G
Urea	Nacalai Tesque	35940-65

**Critical commercial assays**		

Bicinchoninic acid (BCA) protein assay kit	TAKARA	T9300A

**Deposited data**		

CAD data of chamber frame for microscopic image acquisition of thick slices	This paper	Data S1
CAD data of stage adaptor for microscopic image acquisition of thick slices	This paper	Data S2

**Experimental models: Cell lines**		

HEK293T	RIKEN BioResource Research Center	RCB2202

**Experimental models: Organisms/strains**		

Common marmoset (allithrix jacchus)	CLEA Japan	N/A
Mouse: C57BL/6J	Nihon SLC	C57BL/6JJmsSlc
Mouse: *Pvalb*^*tm1(cre)Arbr*^	The Jackson Laboratory	Stock No: 008069
Mouse: PV/myristoylation-EGFP-low-density lipoprotein receptor C-terminal BAC transgenic mice	Kameda et al. (2012)	N/A

**Oligonucleotides**		

Primer: (GGS)2-APEX2: 5′-GGTGGTTCCGGTGGTTCCGGAAAGTCTTACCCAACTGT-3′	This paper	N/A
Primer: BamHI-kozak-EGFP: 5′-AAAAGGATCCGCCACCATGGTGAGCAAGGG-3′	This paper	N/A
Primer: EGFP-(GGS)2: 5′-GGAACCACCGGAACCACCCTTGTACAGCTCGTCCATGC-3′	This paper	N/A
Primer: mScarlet-stop-EcoRI: 5′-TTTTGAATTCTTACTTGTACAGCTCGTCCATGC-3′	This paper	N/A
Primer: Pstl-kozak-mScarlet: 5′-AAAACTGCAGGCCACCATGGTGAGCAAGGGCGAGGC-3′	This paper	N/A
Primer: SalI-APEX2: 5′-TTTTGTCGACTTAGGCATCAGCAAACCCAA-3′	This paper	N/A

**Recombinant DNA**		

pAAV2-DEST(f)	Sohn et al. (2017)	N/A
pAAV2-SynTetOff-BBS	This paper	N/A
pAAV2-SynTetOff-EGFP-APEX2	This paper	N/A
pAAV2-SynTetOff-FLEX-mScarlet	This paper	N/A
pBSIISK-FLEX-mScarlet-BGHpA	This paper	N/A
pBSIISK-hFLEX	Sohn et al. (2017)	N/A
pBSIISK-hFLEX-BGHpA	This paper	N/A
pcDNA3-Connexin43-GFP-APEX2	Addgene	#49385
pENTR1A-SV40LpA-tTAad-SYN-insulator-TRE-GFP-BGHpA	Sohn et al. (2017)	N/A
pENTR1A-SynTetOff-BBS	This paper	N/A
pmScarlet_C1	Addgene	#85042

**Software and algorithms**		

Adobe Photoshop CS6	Adobe	N/A
CANVAS X DRAW	ACD systems	ver. 6.0
Dragonfly software	Object Research System	ver. 2020.2.0.941
EZR	Jichi Medical University, Kanda, 2013	ver. 1.41
GraphPad prism 8	GraphPad Software	N/A
Huygens essential software	Scientific Volume Imaging	ver. 18.10.0p8
ImageJ	National Institutes of Health	ver. 1.52v
Imaris software	Bitplane	ver. 9.0.0
Leica application suite X	Leica Microsystems	LAS X, ver. 3.5.5.19976

**Other**		

1× objective lens	Leica Microsystems	PLANAPO, 10450028
10× objective lens	Leica Microsystems	HCX PL APO 10×/0.40 CS
10× objective lens	Olympus	UPlanApo ×10/0.40
16× multi-immersion objective lens	Leica Microsystems	HC FLUOTAR 16×/0.60 IMM CORR VISIR
20× multi-immersion objective lens	Leica Microsystems	HC PL APO 20×/0.75 IMM CORR CS2
25× water-immersion objective lens	Leica Microsystems	HC FLUOTAR L 25×/0.95 W VISIR
3D printing service	DMM.make	https://make.dmm.com
3D-printer	Keyence	AGILISTA-3200
40× objective lens	Olympus	UPlanApo ×40/0.85
63× oil-immersion objective lens	Leica Microsystems	HC PL APO 63×/1.40 Oil CS2
Amicon® Ultra-15, Ultracel-3K	Merck Millipore	UFC900324
Amicon® Ultra-15, Ultracel-30K	Merck Millipore	UFC903024
Blu-Tak®	Bostik	N/A
Carbon steel blades	Feather	FA-10B
Cooled CCD camera	QImaging	Rolera-XR
Copy stand	LPL	CS-A4 L18142
Digital microscope color camera	Olympus	DP72
Digital microscope color camera	Olympus	DP74
Digital single lens reflex camera	Nikon	D7200
External fluorescence light source	Leica Microsystems	EL6000
FIB-SEM system	Carl Zeiss Microscopy	Crossbeam 540
Freezing microtome	Leica Microsystems	SM2000R
GFP filter cube	Leica Microsystems	excitation filter: 470 ± 20 nm, emission filter: 525 ± 25 nm
LED tracing board	Trytec	A4-500
Light microscope	Olympus	BX-51
Microsyringe pump	KD Scientific	Legato 130
OCT compound	Sakura Finetek	4583
Picospritzer III	Parker Hannifin	N/A
Spectrofluorometer	Perkin Elmer	Enspire 2300
Stereomicroscope	Leica Microsystems	M205C
Stereotaxic apparatus	Narishige	SR50
Stereotaxic apparatus	Narishige	SR-6C-HT
Superfrost APS-coated micro slide glass	Matsunami Glass	APS-01
TCS SP8	Leica Microsystems	N/A
Transmission electron microscope	Hitachi	H-7650
Transmitted light base	Leica Microsystems	TL RCI™
Ultramicrotome	Leica Microsystems	Ultracut UCT
Upright microscope	Olympus	BX51
UV transparent 96-well plate	Greiner Bio-One	655801
Vibrating tissue slicer	Dosaka EM	Linear PRO7N

### Resource availability

#### Lead contact

Further information and requests for resources should be directed to and will be fulfilled by the Lead Contact, Hiroyuki Hioki (h-hioki@juntendo.ac.jp).

#### Materials availability

All unique reagents generated in this study are available from the Lead Contact with a completed Material Transfer Agreement.

### Experimental model and subject details

#### Animals

All animal experiments involving animal care, surgery, and sample preparation were approved by the Institutional Animal Care and Use Committees of Osaka University (Approval No. 300150), Juntendo University (Approval No. 2020087, 2020088), and Kyoto University (Approval No. Med Kyo 20031) and conducted in accordance with Fundamental Guidelines for Proper Conduct of Animal Experiments by the Science Council of Japan (2006). All efforts were made to minimize animal suffering and the number of animals used.

Eight- to twelve-week-old male C57BL/6J (Nihon SLC), *PV*^*Cre*^ heterozygous (*Pvalb*^*tm1(cre)Arbr*^, The Jackson Laboratory Stock No: 008069) (Hippenmeyer et al., 2005), and PV/myristoylation-EGFP-low-density lipoprotein receptor C-terminal BAC transgenic mice (PV-FGL mice) (Kameda et al., 2012) under specific pathogen-free conditions were used. The mice were maintained under a 12/12 hr light/dark cycle (light: 08:00–20:00) with *ad libitum* access to food and water. Mouse genotypes were determined by polymerase chain reaction (PCR) analysis as described previously (Kameda et al., 2012).

Four young adult (14–15 months old) male or female common marmosets (Callithrix jacchus; body weight, 280–400 g; bred either in CLEA Japan or in our laboratory) were housed in their home cages under a 14/10 hr light/dark cycle (light: 07:00–21:00). Each cage had a wooden perch, a food tray, and an automatic water dispenser. The animals were fed twice a day with solid food (CMS-1, CLEA Japan). Water was provided *ad libitum*.

#### HEK293T cell culture

HEK293T cells (RCB2202, RIKEN BioResource Research Center) were cultured in Dulbecco's modified Eagle's medium (11965-092, Thermo Fisher Scientific) containing 10% fetal bovine serum (173012, Sigma-Aldrich), 2 mM L-glutamine (25030-081, Thermo Fisher Scientific), 1× MEM Non-Essential Amino Acid (11140-050, Thermo Fisher Scientific), and 1× Penicillin-Streptomycin (15070-063, Thermo Fisher Scientific) in a 37°C incubator with 5% CO_2_. Before reaching 70% confluence, the cells were passaged using TrypLE Express (12605-010, Thermo Fisher Scientific).

### Method details

#### Preparation of tissue slices

Mice were deeply anesthetized by intraperitoneal injections of sodium pentobarbital (200 mg/kg; Somnopentyl, Kyoritsu Seiyaku) and perfused transcardially with 20 mL of 5 mM phosphate-buffered 0.9% saline (PBS; pH 7.4) at 4°C, followed by the same volume of 4% PFA (1.04005.1000, Merck Millipore) or 4% PFA containing various concentrations (0.02, 0.2, 1, or 2%) of GA (17003-92, Nacalai Tesque) in 0.1 M phosphate buffer (PB; pH 7.4) at 4°C. The brains of the animals were removed and postfixed in the same fixatives overnight at 4°C. After embedding in 4% agar (01028-85, Nacalai Tesque) in PBS, coronal or sagittal slices of 1-mm thickness were cut with a vibrating tissue slicer (Linear PRO7N, Dosaka EM).

The marmosets were deeply anesthetized by intramuscular injections of ketamine (60 mg/kg; Ketalar, Daiichi Sankyo Propharma) and intraperitoneal injections of sodium pentobarbital (80 mg/kg). The fixation and preparation of the tissue slices of marmoset brains were the same as those used for the mice, except that their perfusion with 300 mL of PBS containing 1.0 unit/mL heparin (224122458, Mochida Pharmaceutical) followed by the same volume of 4% PFA or 4% PFA containing GA (0.2% or 1%) in 0.1 M PB.

#### Tissue clearing

The schedule for tissue clearing with Sca*l*eSF is described in Figure 2A. Brain slices were permeabilized with Sca*l*eS0 solution for 2 hr at 37°C, washed twice with PBS(–) (27575-31, Nacalai Tesque) for 15 min at 20–25°C, and cleared with Sca*l*eS4 solution for 8–12 hr at 37°C. We treated brain slices with Sca*l*eS4 solution for 12 hr in the data shown in this paper. The formula for Sca*l*eS0 solution was 20% (w/v) sorbitol (06286-55, Nacalai Tesque), 5% (w/v) glycerol (G9012, Sigma-Aldrich), 1 mM methyl-β-cyclodextrin (M1356, Tokyo Chemical Industry), 1 mM γ-cyclodextrin (037-10643, Wako Pure Chemical Industries), and 3% (v/v) dimethyl sulfoxide (DMSO) (13407-45, Nacalai Tesque) in PBS(–), and that for Sca*l*eS4 solution was 40% (w/v) sorbitol, 10% (w/v) glycerol, 4 M urea (35940-65, Nacalai Tesque), 0.2% (w/v) Triton X-100 (35501-15, Nacalai Tesque), and 25% (v/v) DMSO in distilled deionized water (DDW) (Miyawaki et al., 2016).

The optical clearing methods of brain slices with CUBIC, PACT, Sca*l*eSQ(0) and SeeDB followed the protocol as below. Considering 1-mm-thick slices clearing, incubation time of each method was adjusted accordingly.

##### CUBIC

Brain slices were incubated in 50% CUBIC-L (T3740, Tokyo Chemical Industry) in DDW for 1 hr and CUBIC-L for 12 hr at 37°C. After washing twice with PBS(−) for 30 min, the delipidated slices were cleared by incubations in 50% CUBIC-R + (M) (T3741, Tokyo Chemical Industry) in DDW and CUBIC-R + (M), each for 4 hr at 20–25°C (Tainaka et al., 2018).

##### PACT

Brain slices were incubated at 4°C overnight in A4P0 hydrogel (4% [w/v] acrylamide [161-0140, Bio-Rad] and 0.25% 2,2′-Azobis[2-(2-imidazolin-2-yl)propane] dihydrochloride [VA-044, Wako Pure Chemical Industries] in PBS(−)). The slices were vacuum degassed for 10 min, placed under nitrogen for 10 min, and incubated at 37°C for 3 hr to initiate tissue-hydrogel hybridization. After washing twice with PBS(−) for 15 min at 20–25ºC, the slices were incubated at 37°C for 24 hr in 8% (w/v) sodium dodecyl sulfate (31607-65, Nacalai Tesque) in PBS(−) (Yang et al., 2014).

##### ScaleSQ(0)

Sca*l*eSQ(0) solution (9.1 M urea and 22.5% [w/v] sorbitol in DDW) was prepared and stored at 37°C. Brain slices were cleared by an incubation in the solution at 37°C for 2 hr (Hama et al., 2015).

##### SeeDB

20%, 40%, 60%, 80%, 100% (w/v) and 80.2% (w/w) fructose (F0127, Sigma-Aldrich) were dissolved in DDW and α-thioglycerol (M1753, Sigma-Aldrich) was added to give a final concentration of 0.5% (SeeDB [standard] solutions). Brain slices were serially incubated in the 20%, 40% and 60% (w/v) fructose solution, each for 4 hr, and then incubated in the 80% and 100% (w/v) fructose solution, each for 12 hr. Afterward, the slices were cleared in the 80.2% [w/w] fructose solution (SeeDB) for 24 hr. All incubations were performed at 20–25°C (Ke et al., 2013).

#### Observation and measurement of macroscopic structures

Transmission images of mouse brain slices were acquired with a stereomicroscope (M205C, Leica Microsystems) equipped with a 1× objective lens (PLANAPO, working distance [WD] = 65 mm, Leica Microsystems), a transmitted light base (TL RCI, Leica Microsystems), and a digital single lens reflex camera (D7200, Nikon). Marmoset brain slices were placed on a LED tracing board (A4-500, Trytec) and imaged with the digital single lens reflex camera mounted on a copy stand (CS-A4 L18142, LPL). Fluorescence images of the marmoset brains were acquired with the stereomicroscope equipped with an external fluorescence light source (EL6000, Leica Microsystems), a GFP filter cube (excitation filter: 470 ± 20 nm, emission filter: 525 ± 25 nm, Leica Microsystems), and a cooled CCD camera (Rolera-XR, QImaging). Brain samples were placed on graph paper with a patterned background (ruled into 1-mm squares).

To assess tissue expansion or shrinkage caused by tissue clearing, brain-slice areas were measured with ImageJ (ver. 1.52v, National Institutes of Health) (Schneider et al., 2012). Linear expansion values were determined based on the square root of the changes in the brain-slice areas.

#### Transmission measurements

Light transmittance of brain slices was determined with a spectrofluorometer (Enspire 2300, Perkin Elmer). Coronal brain slices at the level of the S1 were used. Brain slices cleared with Sca*l*eSF or Sca*l*eSQ(0), or stored in PBS(−) were transferred onto UV transparent 96-well plates (655801, Greiner Bio-One) to measure absorbance of the tissues. The absorbance (A) was converted to percent transmittance (%T) using an equation derived from Lambert-Beer's law: A = 2 – log10 (%T).

To assess clearing efficacy of Sca*l*eSF in gray and white matter regions, mean gray values of the gray and white matter of the cerebral cortex in brain slices cleared with Sca*l*eSF were measured with Image J software. Transmission images of Sca*l*eSF-treated brain slices were acquired with the stereomicroscope.

#### Imaging chamber and tissue mounting

A customizable 3D-printed imaging chamber that enabled reliable mounting of cleared tissues was designed for imaging with CLSM (Figure S3). The chamber consisted of the chamber frame, bottom coverslip, and microscope stage adaptors (Figure S3A). The frames and adaptors were designed according to the size and thicknesses of brain slices and printed from a rigid acrylic resin, AR-M2 (Keyence), using a 3D-printer (AGILISTA-3200, Keyence) by DMM.make (https://make.dmm.com) (Data S1 and S2). The frames were glued to the bottom coverslips (Matsunami Glass). Optically cleared tissues were mounted on the coverslips and embedded in 1.5% Agarose (L03, TaKaRa Bio) in Sca*l*eS4D25(0) solution (Sca*l*eS4 gel) (Miyawaki et al., 2016). Tissues were coverslipped and left at 4°C until the gel solidified. The imaging chambers were attached to the microscope stage adaptors to mount on microscope stages (Figures S3B and S3C) or attached to petri dishes with Blu-Tak (Bostik) and immersed in Sca*l*eS4 solution (Figure S3D).

#### Confocal laser scanning microscope

3D image stacks were acquired with a TCS SP8 CLSM (Leica Microsystems). A 16× multi-immersion objective lens (HC FLUOTAR 16×/0.60 IMM CORR VISIR, numerical aperture [NA] = 0.60, WD = 2.5 mm, Leica Microsystems) was used for imaging the optically cleared brain slices (1-mm thick). 10× air (HCX PL APO 10×/0.40 CS, NA = 0.40, WD = 2.20 mm, Leica Microsystems), 20× multi-immersion (HC PL APO 20×/0.75 IMM CORR CS2, NA = 0.75, WD = 0.66 mm, Leica Microsystems), 25× water-immersion (HC FLUOTAR L 25×/0.95 W VISIR, NA = 0.95, WD = 2.40 mm, Leica Microsystems), and 63× oil-immersion (HC PL APO 63×/1.40 Oil CS2, NA = 1.40, WD = 0.14 mm, Leica Microsystems) objective lenses were used for imaging the re-sections (40- or 50-μm thick). Sections were mounted with PBS or 75% glycerol in PBS (Hirabayashi et al., 2018). DAPI, EGFP, and mScarlet were excited by 405-, 488-, and 552-nm lasers, and their emissions were collected through 410–480, 495–525, and 560–700 nm emission prism windows, respectively.

#### Transmission electron microscopy

Sample preparation and imaging of cleared brain slices with TEM were carried out as described previously (Hama et al., 2015), with minor modifications. Briefly, after restoration of cleared brain slices by washing with PBS(−), 1-mm cubes were excised from the brain slices with carbon steel blades (FA-10B, Feather). 1-mm cubes were also prepared from brain slices stored in PBS at 4°C during clearing procedures. The cubes and re-sections (50-μm thick) prepared from 1-mm-thick slices were osmicated with 1% OsO_4_ (25746-06, Nacalai Tesque) in 0.1 M PB, dehydrated with a gradient series of ethanol (50, 70, 90, 99, and 100%) followed by propylene oxide (29223-55, Nacalai Tesque), and embedded in an Epon 812 mixture (a mixture of Luveak-812 [20829-05, Nacalai Tesque], Luveak-DDSA [14423-95, Nacalai Tesque], Luveak-MNA [14424-85, Nacalai Tesque], and Luveak-DMP-30 [14425-75, Nacalai Tesque]) or Durcupan (44610, Sigma-Aldrich). To test the accelerator Luveak-DMP-30 for the permeability of the Epon 812 mixture into the tissues, resin polymerization was initiated after pre-incubation with an Epon 812 mixture that did not contain the accelerator (modified Epon method). After polymerization of the resin, ultrathin sections (70-nm thick) were cut with an ultramicrotome (Ultracut UCT, Leica Microsystems). The sections were stained with 1% uranyl acetate and 1% lead citrate, and observed under a TEM (H-7650, Hitachi) at 80 kV. We acquired digital photographs of presynaptic axonal terminals, which contained synaptic vesicles and formed synapses onto dendritic structures, at a resolution of 1.2 nm/pixel.

To evaluate ultrastructural preservation, the plasma membrane of the presynaptic structures was outlined with a graphic software (CANVAS X DRAW, ACD systems). Membrane continuities of presynaptic structures of >90%, 50–90%, 10–50%, and <10% were scored as 4, 3, 2, and 1, respectively.

#### Scanning electron microscopy combined with focused ion beam

We performed 3D imaging of synaptic structures by FIB-SEM technique as described previously (Sonomura et al., 2013), with minor modifications. In brief, brain sections (50-μm thick) were osmicated with 2% OsO_4_ in 0.1 M PB, counterstained with 1% uranyl acetate for 2 hr, and stained in lead aspartate solution at 60°C for 30 min. After dehydration with a gradient series of ethanol (60, 70, 80, 90, 99, and 100%) and propylene oxide, the sections were flat-embedded in the Epon 812 mixture. The regions that contained targeted structures were excised with carbon steel blades from the embedded sections, mounted on aluminum stubs, and examined with a FIB-SEM system (Crossbeam 540, Carl Zeiss Microscopy). Using the FIB of 30 kV and 3 nA, a surface layer of 10-nm thickness was milled at each sectioning. Following the removal of each layer, the freshly exposed surface was imaged with the SEM using the back-scattered electron detector at a magnification of 10 nm/pixel. The acceleration voltage of the imaging beam was 1.5 kV with a beam current of 1 nA and a dwell time of 13.6 μs/pixel.

#### Measurement of solubilized protein in clearing reagents

To evaluate protein preservation during clearing procedures, total amount of solubilized protein in clearing reagents was measured. Five 1-mm-thick brain slices fixed with 4% PFA containing 1% GA were divided into left and right sides at the midline, either side was cleared with Sca*l*eSF or SeeDB, and then weighted. The respective clearing solutions were collected, pooled and a protease inhibitor cocktail (P8849, Sigma-Aldrich) was added to the pooled solution. The solutions were then diafiltrated to approximately 2,500,000-fold dilution in PBS to remove solutes and concentrated to 300 μL with 3 kDa molecular weight cutoff (MWCO) filter units (UFC900324, Merck Millipore). The protein concentrations of the diafiltrated and concentrated solutions were measured with a bicinchoninic acid (BCA) protein assay kit following the manufacturer's “low-concentration” protocol (T9300A, TAKARA). The amount of solubilized protein per the weight of brain slices was calculated and normalized to that in Sca*l*eSF.

#### AAV vector construction and production

pAAV2-SynTetOff-EGFP-APEX2 was constructed as follows. The GFP sequence of pENTR1A-SV40LpA-tTAad-SYN-insulator-TRE-GFP-BGHpA (Sohn et al., 2017) was replaced with a multiple cloning site, which contained BamHI-BglII-SalI restriction sites. The resultant entry vector pENTR1A-SynTetOff-BBS was reacted with pAAV2-DEST(f) (Sohn et al., 2017) by homologous recombination with LR clonase II (11791020, Thermo Fisher Scientific) to generate pAAV2-SynTetOff-BBS. A DNA fragment coding an EGFP-APEX2 fusion protein was generated by overlapping PCR. A sequence coding for a peptide linker (Gly-Gly-Ser)_2_ was inserted between the two protein domains. The coding sequence of APEX2 was amplified from pcDNA3-Connexin43-GFP-APEX2 (#49385, Addgene) (Lam et al., 2015). The restricted product was inserted into pAAV2-SynTetOff-BBS through the BamHI/SalI sites, resulting in pAAV2-SynTetOff-EGFP-APEX2. For the construction of pAAV2-SynTetOff-FLEX-mScarlet, the BGHpA sequence was digested through the MluI/NotI sites of pAAV2-SynTetOff-GFP and inserted into the MluI/NotI sites of pBSIISK-hFLEX (Sohn et al., 2017), resulting in pBSIISK-hFLEX-BGHpA. The coding sequence of mScarlet was amplified from pmScarlet_C1 (#85042, Addgene) (Bindels et al., 2017) and inserted into pBSIISK-hFLEX-BGHpA through the PstI/EcoRI sites to generate pBSIISK-FLEX-mScarlet-BGHpA. The pBSIISK-FLEX-mScarlet-BGHpA was then digested with BamHI/SphI and ligated into the corresponding sites of pAAV2-SynTetOff-BBS, yielding pAAV2-SynTetOff-FLEX-mScarlet. The following primers were used for PCR amplification: BamHI-kozak-EGFP: 5′-AAAAGGATCCGCCACCATGGTGAGCAAGGG-3′, EGFP-(GGS)_2_: 5′-GGAACCACCGGAACCACCCTTGTACAGCTCGTCCATGC-3′, (GGS)_2_-APEX2: 5′-GGTGGTTCCGGTGGTTCCGGAAAGTCTTACCCAACTGT-3′, SalI-APEX2: 5′-TTTTGTCGACTTAGGCATCAGCAAACCCAA-3′, Pstl-kozak-mScarlet: 5′-AAAACTGCAGGCCACCATGGTGAGCAAGGGCGAGGC-3′, and mScarlet-stop-EcoRI: 5′-TTTTGAATTCTTACTTGTACAGCTCGTCCATGC-3′.

AAV vector particles were produced and purified as described previously (Sohn et al., 2017; Takahashi et al., 2021). Briefly, pAAV2-SynTetOff-EGFP-APEX2 or pAAV2-SynTetOff-FLEX-mScarlet and two helper plasmids were co-transfected into HEK293T cells using polyethylenimine (23,966, Polysciences). Virus particles were purified from the cell lysate and supernatant by ultracentrifugation with OptiPrep (1114542, Axis-Shield) and concentrated by ultrafiltration with Amicon Ultra-15 (UFC903024, Merck Millipore). The infectious titer of the AAV vector (IFU/mL) was determined by quantitative PCR (qPCR) with HEK293T cells infected with the purified AAV vectors. The physical titer of the AAV vector (genome copies (gc)/mL) was measured by qPCR with the purified virus solution. The solution was stored in aliquots at −80°C until use.

#### Virus injection

Virus injection into mouse brains was conducted as described previously (Okamoto et al., 2020, 2021), with some modifications. Briefly, mice were deeply anesthetized with intraperitoneal injections of medetomidine (0.3 mg/kg; Domitor, Zenoaq), midazolam (4 mg/kg; Dormicum, Astellas Pharma), and butorphanol (5 mg/kg; Vetorphal, Meiji Seika Pharma) and placed in a stereotaxic apparatus (SR50, Narishige). Subsequently, 0.2 μL of the virus solution (AAV2/1-SynTetOff-EGFP-APEX2: 1.32 × 10^11^ IFU/mL, AAV2/1-SynTetOff-FLEX-mScarlet: 1.8 × 10^13^ gc/mL) was pressure-injected into the M1, S1, and CPu through a glass micropipette attached to Picospritzer III (Parker Hannifin). The injection coordinates were as follows: M1: 1.0 mm anterior to the bregma, 1.2 mm lateral to the bregma, and 0.8 mm ventral to the brain surface; S1: 2.0 mm lateral to the bregma, 0.5 mm ventral to the brain surface; and CPu: 0.5 mm anterior to the bregma, 2.0 mm lateral to the bregma, and 2.5 mm ventral to the brain surface. The mice were recovered from anesthesia with intraperitoneal injections of atipamezole (1.5 mg/kg; Antisedan, Zenoaq) and maintained under regular health checks for one to six weeks.

Virus injection into marmoset brains was performed as follows (Watakabe et al., 2015). All surgical procedures were conducted under aseptic conditions. Animals were anesthetized with intramuscular injections of ketamine (15 mg/kg) and medetomidine (50 μg/kg) and pre-medicated with intramuscular injections of atropine (40 μg/kg; Nipro ES Pharma), ampicillin (25 mg/kg; Viccillin, Meiji Seika pharma), and dexamethasone (80 μg/kg; Decadron, Aspen Japan), as well as subcutaneous injections of a lactated Ringer's solution (10 mL/kg; Solulact, Terumo) at 37°C. The animals were placed under deep anesthesia with isoflurane (1–2% in oxygen, Pfizer) inhalation. The head was fixed to a stereotaxic apparatus (SR-6C-HT, Narishige). Heart rate, percutaneous oxygen saturation (SpO_2_), and rectal temperature were continuously monitored. A small hole was made in the skull with a dental drill. A glass micropipette with a tip diameter of 50 μm was filled with the virus solution (AAV2/1-SynTetOff EGFP-APEX2, 1.32 × 10^11^ IFU/mL). After incision of the dura, the pipette was slowly lowered to the target depth and fixed for 3 min, and 0.15 μL of the virus solution was injected at a rate of 75 nL/min with a microsyringe pump (Legato 130, KD Scientific). The micropipette was held in place for 5 min and then extracted. The injection coordinates were as follows: 9.25 mm, 8.2 mm, 7.2 mm, and 6.15 mm anterior to the interaural line, 5.0 mm lateral to the midline, and 1.0 mm ventral to the brain surface. After the topical administration of gentamicin (Nichi-Iko Pharmaceutical), the head skin was closed by suturing. The animals were then received intramuscular injections of dexamethasone (80 μg/kg), diclofenac sodium (1.0 mg/kg, 11147700J1057, Novartic Japan), and ampicillin (25 mg/kg), as well as subcutaneous injections of lactated Ringer's solution (10 mL/kg) at 37°C. After surgery, the animals were recovered from anesthesia with intramuscular injections of atipamezole (40 to 480 μg/kg), and ampicillin was administered for two days (25 mg/kg/day). The animals were maintained under regular health checks for six weeks.

#### DAB-Ni^2+^ labeling by APEX2

The effects of Sca*l*eSF clearing on peroxidase activity of APEX2 were assessed by DAB polymerization. The brains were fixed with 4% PFA containing 0.2% GA seven to ten days after the injections of the AAV2/1-SynTetOff-EGFP-APEX2 vector into the S1. The brain slices (1-mm thick) expressing an EGFP-APEX2 fusion protein were cleared with Sca*l*eSF. EGFP fluorescence in the slices was examined under the fluorescence stereomicroscope. After deSca*l*ing with PBS(−), the slices were cryoprotected in 30% sucrose in 0.1 M PB at 4°C, embedded in OCT compound (4583, Sakura Finetek), and frozen in liquid nitrogen-cooled isopentane. The slices were then cut into 40-μm-thick sections on a freezing microtome (SM2000R, Leica Microsystems). Following CLSM imaging, the sections were permeabilized with PBS containing 0.3% Triton X-100 (0.3% PBS-X) and developed in 0.05% DAB·4HCl (347-00904, Dojindo), 25 mM nickel ammonium sulfate (24217-82, Nacalai Tesque), and 0.0003% H_2_O_2_ in 50 mM Tris-HCl (pH 7.6) (DAB-Ni^2+^ solution).

#### DAB-Ni^2+^ labeling by APEX2/BT-GO reaction

DAB polymerization in brain slices cleared with Sca*l*eSF was enhanced with APEX2/BT-GO (biotinylated tyramine-glucose oxidase) reaction, in which biotin molecules were deposited with TSA reaction using peroxidase activity of APEX2. Brains were fixed with 4% PFA containing 0.2% GA and cut into 1-mm-thick slices. EGFP-APEX2 expression in brain slices was examined as described above. The slices were then permeabilized for 4 hr with 0.2% PBS-X containing 2% bovine serum albumin (BSA) (01863-77, Nacalai Tesque), washed thrice with 0.1 M PB, and incubated for 4 hr in a BT-GO reaction mixture that contained 25 μM biotinylated tyramine and 3 μg/mL glucose oxidase (16831-14, Nacalai Tesque) in 2% BSA in 0.1 M PB (Furuta et al., 2009; Kuramoto et al., 2009; Okamoto et al., 2021). TSA reaction was initiated by adding 2 mg/mL of ß-D-glucose (049-31165, Wako Pure Chemical Industries) into the reaction mixture and proceeded for 2 hr. The brain slices were washed with PBS(−), fixed with 4% PFA in 0.1 M PB overnight at 4°C, and cleared with Sca*l*eSF. Cryosections (40- or 50-μm thick) or vibratome sections (50-μm thick) were prepared from deSca*l*ed slices as described above. Some of the sections were counterstained with DAPI (1 μg/mL, D1306, Thermo Fisher Scientific) in PBS for 2 hr on ice. The sections were then reacted with avidin-biotinylated peroxidase complex (ABC) (1:50 diluted in PBS, PK-6100, Vector Laboratories) in PBS containing 2% BSA overnight at 4°C and developed in DAB-Ni^2+^ solution on ice. CLSM imaging was performed prior to the ABC reaction.

#### Bright-field microscopy

Bright-field images of tissue sections were obtained with a light microscope (BX-51, Olympus) equipped with dry objectives (10× UPlanApo, NA = 0.40, WD = 3.1 mm, Olympus; 40× UPlanApo, NA = 0.85, WD = 0.2 mm, Olympus) and a CCD camera (DP72 or DP74, Olympus). DAB-Ni^2+^-labeled sections were mounted onto glass slides (Superfrost micro slide glass APS-coated, Matsunami Glass) and coverslipped with 50% glycerol, 2.5% 1,4-diazabicyclo[2.2.2]octane (DABCO) (049-25712, Wako Pure Chemical Industries), and 0.02% sodium azide (31233-55, Nacalai Tesque) in PBS.

#### Image processing

Three-dimensional renderings of CLSM image stacks were created with Imaris software (ver. 9.0.0, Bitplane). Images appearing in Figures 1F–1J were deconvoluted with Huygens Essential software (ver. 18.10.0p8, Scientific Volume Imaging) before the rendering process. Maximum intensity projection and orthogonal images were created using Leica Application Suite X (LAS X, ver. 3.5.5.19976, Leica Microsystems) and Imaris software. Three-dimensional reconstruction of FIB-SEM datasets was conducted using Dragonfly software (ver. 2020.2.0.941, Object Research System). The global brightness and contrast of the images were adjusted with ImageJ, Adobe Photoshop CS6 (Adobe), and CANVAS X DRAW.

### Quantification and statistical analysis

Data are represented as means ± standard deviations (SDs). The exact values of n are indicated in the corresponding figure legends. For comparisons between groups, unpaired Student's t-test (Figures 2D and S2B) or Mann-Whitney U test (Figure S5F) was used. For comparisons among independent groups, one-way analysis of variance (ANOVA) (Figure 3B), Kruskal–Wallis test followed by Steel–Dwass *post hoc* test (Figures 4B and S5E), or Kruskal–Wallis test (Figure S4C) was used. For comparisons between groups over time, two-way repeated measures ANOVA followed by Tukey *post hoc* test was used (Figure S1B). The equality of probability distributions was assessed using Kolmogorov–Smirnov test. All tests were two-sided. Statistical analyses were conducted using EZR (ver. 1.41, Saitama Medical Center, Jichi Medical University) (Kanda, 2013) and GraphPad Prism 8 (GraphPad Software). Statistical significance was set at *P* < 0.05.

## Data Availability

• All data reported in this paper will be shared by the lead contact upon request. • All original code is available in this paper's supplemental information. • For any additional questions or information please contact the lead contact.

## Supplemental information

Document S1. Figures S1–S7

Data S1. Chamber frame of customizable 3D-printed imaging chambers designed for 1-mm-thick slice imaging under CLSM, related to Figure 2

Data S2. Adaptor for customizable 3D-printed imaging chambers designed for 1-mm-thick slice imaging under CLSM, related to Figure 2
