# Supplementary material for: Multi-scale light microscopy/electron microscopy neuronal imaging from brain to synapse with a tissue clearing method, ScaleSF
Source: iScience. 2021 Dec 27;25(1):103601. doi: 10.1016/j.isci.2021.103601 (PMC8786651; doi:10.1016/j.isci.2021.103601)
Supplement: Document S1. Figures S1–S7 [file mmc1.pdf]

iScience, Volume *25*

Supplemental information

Multi-scale light microscopy/electron microscopy neuronal imaging from brain to synapse with a tissue clearing method, Sca*l*eSF

Takahiro Furuta, Kenta Yamauchi, Shinichiro Okamoto, Megumu Takahashi, Soichiro Kakuta, Yoko Ishida, Aya Takenaka, Atsushi Yoshida, Yasuo Uchiyama, Masato Koike, Kaoru Isa, Tadashi Isa, and Hiroyuki Hioki

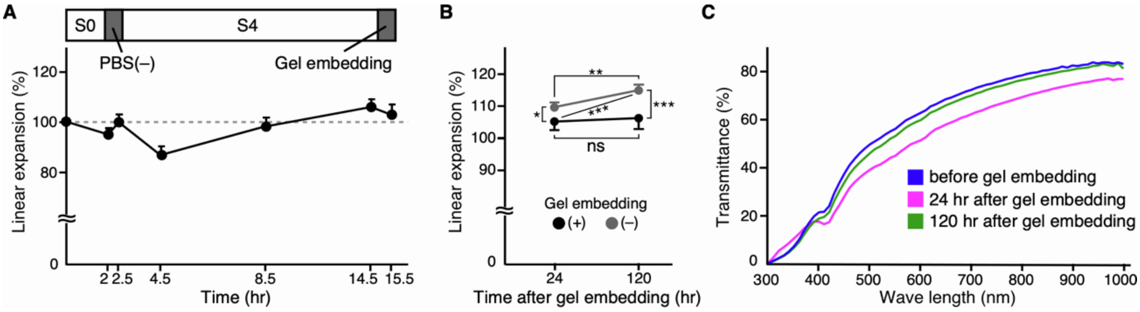

Figure S1. Change in size and transparency of mouse brain slices after embedding with Sca*l*eS4 gel. Related to Figure 2.

(A) Changes in the size of mouse brain slices during Sca*l*eSF treatment (n = 3 brain hemispheres). Data are represented as means ± SDs.

(B) Changes in the size of Sca*l*eSF-treated mouse brain slices with and without Sca*l*eS4 gel embedding (n = 3 brain hemispheres each, Gel embedding, $F_{1,4} = 34.2$, $P = 0.0043$; Time, $F_{1,4} = 83.45$, $P = 0.0008$; Interaction, $F_{1,4} = 42.38$, $P = 0.0029$; two-way repeated measures ANOVA; * $P < 0.05$, ** $P < 0.01$, *** $P < 0.001$; Tukey *post hoc* test). Data are represented as means ± SDs.

(C) Transmission curves of Sca*l*eSF-treated mouse brain slices before, and 24 and 120 hr after Sca*l*eS4 gel embedding (n = 3 brain hemispheres each). The thickness of brain slices is 1 mm. Data are represented as means.

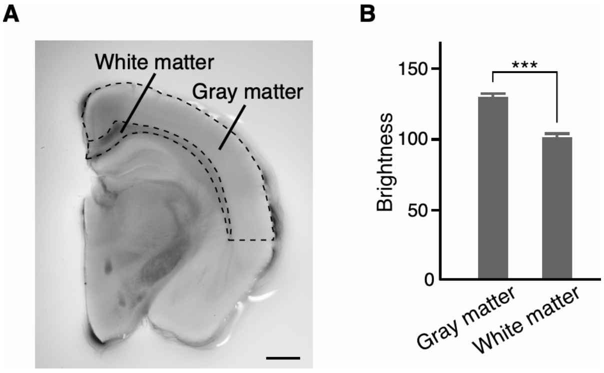

Figure S2. Difference in transparency between the gray and white matter of the cerebral cortex. Related to Figure 2.

(A) A transmission image of 1-mm-thick brain slices treated with Sca*l*eSF. Scale bar, 1 mm. Mean gray values in the areas enclosed by broken lines are measured for quantitative analysis.

(B) Mean gray values in the gray and white matter of the cerebral cortex cleared with Sca*l*eSF (n = 3, t = 12.914, df = 4, $P = 2.07 \times 10^{-4}$, two-tailed unpaired Student's t-test). Data are represented as means ± SDs.

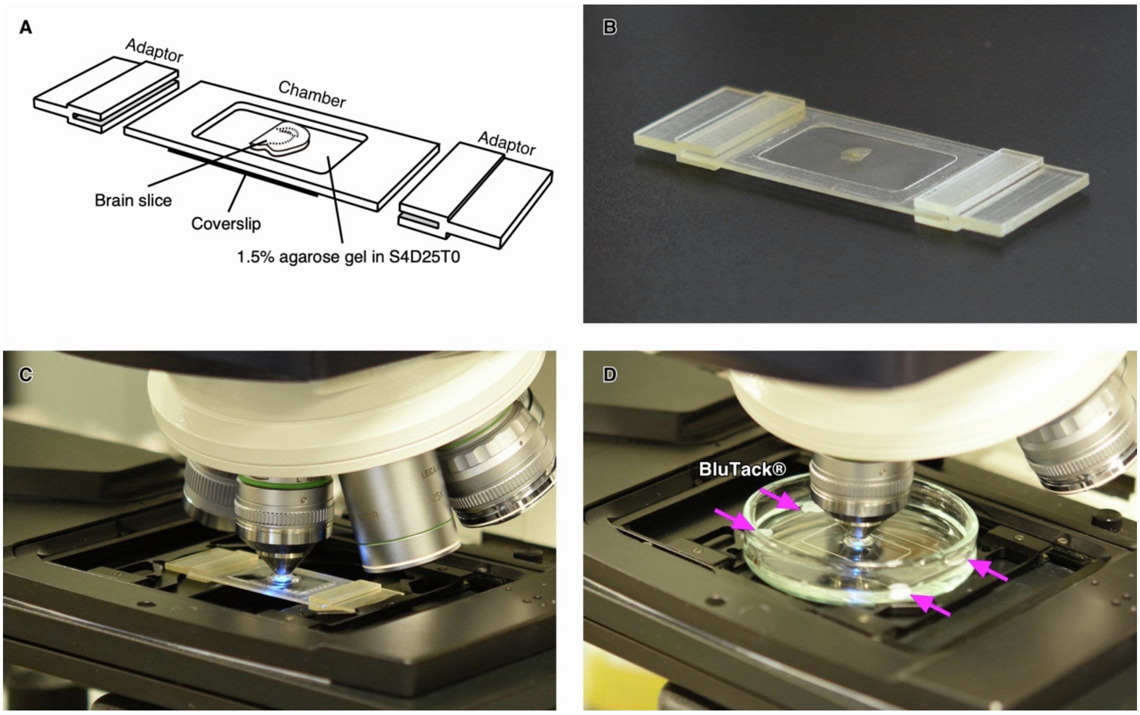

Figure S3. Customizable 3D-printed imaging chambers for Sca*l*eSF-treated tissues. Related to Figure 2.

(A and **B)** A schematic drawing (A) and a picture (B) of a customizable 3D-printed imaging chamber. The imaging chamber comprises a chamber frame, a bottom coverslip, and microscope stage adaptors. Brain slices cleared with Sca*l*eSF are mounted on the bottom coverslip and embedded in 1.5% agarose gel in Sca*l*eS4D25(0) solution (Sca*l*eS4 gel). The frame and adaptors can be customized according to the size and thicknesses of brain slices. The bottom coverslip allows for imaging with inverted microscopes. The imaging chamber enables reliable mounting of cleared tissues.

(C and **D)** Imaging setups with the imaging chamber and an upright CLSM. The imaging chamber mounted on a microscope stage using the microscope stage adaptors (C). The imaging chamber without adaptors is immersed in Sca*l*eS4 solution and mounted on the microscope stage (D). Blu-tack® (arrows) is used to hold the imaging chamber firmly to the glass dish. Note that brain slices in (B–D) are not cleared.

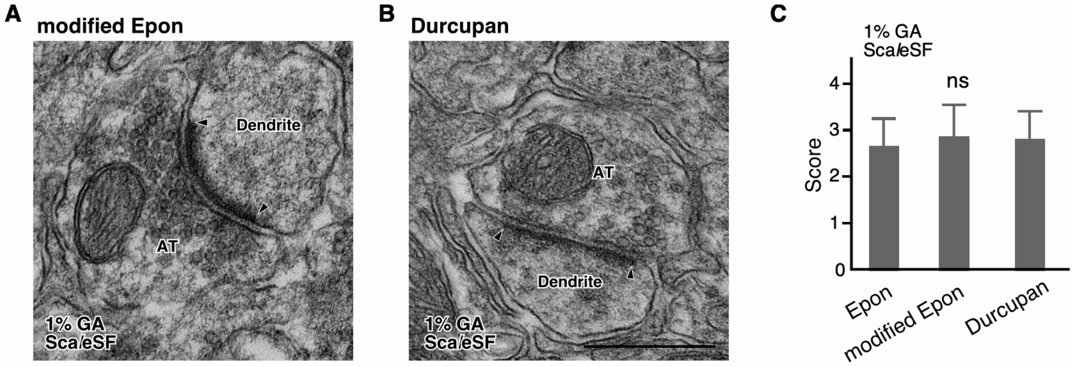

Figure S4. Effects of different epoxy resin embedding methods on ultrastructure preservation in Sca*l*eSF-treated mouse brain slices. Related to Figure 4.

(A and **B)** TEM images of Sca*l*eSF-treated mouse brain slices embedded with the modified Epon method (A) or Durcupan (B). When using the modified Epon method, resin polymerization is initiated after pre-incubation in an epoxy mixture without an accelerator. The brains are fixed with 4% PFA containing 1% GA. AT: axon terminal. Scale bar, 500 nm.

(C) Scoring of membrane continuity of the presynaptic terminals for each condition. Over 90%, 50-90%, 10-50%, and less than 10% membrane continuity of the presynaptic terminals are scored as 4, 3, 2 and 1, respectively. There are no significant differences among the groups (n = 34 synapses, Epon; n = 31 synapses, modified Epon; n = 31 synapses, Durcupan; n = 3 mice for each condition; H = 1.583, df = 2, P = 0.4532, Kruskal-Wallis test). Data are represented as means ± SDs.

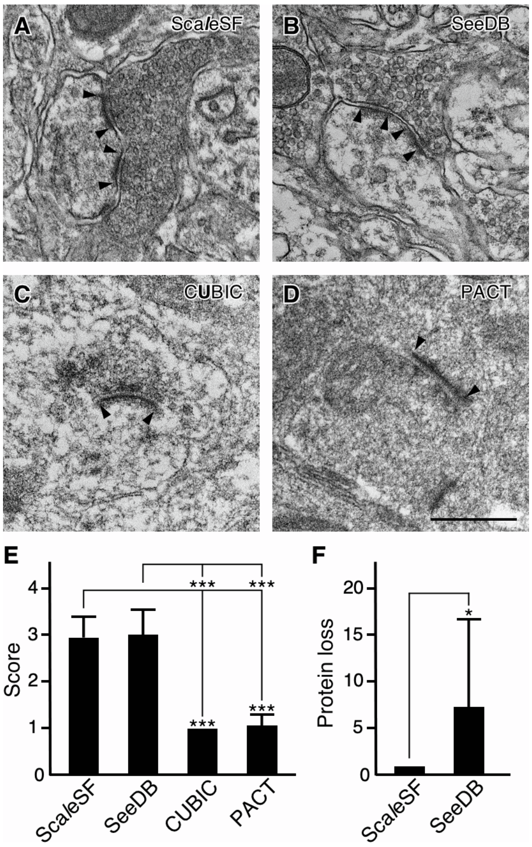

Figure S5. Ultrastructural preservations in GA-fixed brain slices cleared with Sca*l*eSF, SeeDB, CUBIC, and PACT. Related to Figure 4.

(A–D) TEM images of mouse cerebral cortex cleared with Sca*l*eSF (A), SeeDB (B), CUBIC (C), and PACT (D). Mouse brains were fixed with 4% PFA containing 1% GA. Arrowheads indicate postsynaptic membranes. Scale bar, 500 nm.

(E) Scoring of membrane continuity of presynaptic terminals for each condition in (A–D) (n = 34 synapses, Sca*l*eSF; n = 34 synapses, SeeDB; n = 34 synapses, CUBIC; n = 33 synapses, PACT; n = 3 mice for each condition; $H = 99.77$, $df = 3$, $P = 1.74 \times 10^{-21}$, Kruskal–Wallis test; * $P < 0.05$, ** $P < 0.01$, *** $P < 0.001$; Steel–Dwass *post hoc* test). Data are represented as means ± SDs.

(F) The amount of solubilized protein in clearing reagents of Sca*l*eSF and SeeDB (n = 6 for each condition; $U = 6$, $P = 4.76 \times 10^{-2}$, Mann-Whitney U test; * $P < 0.05$). Data are normalized to the solubilized protein in Sca*l*eSF and represented as means ± SDs.

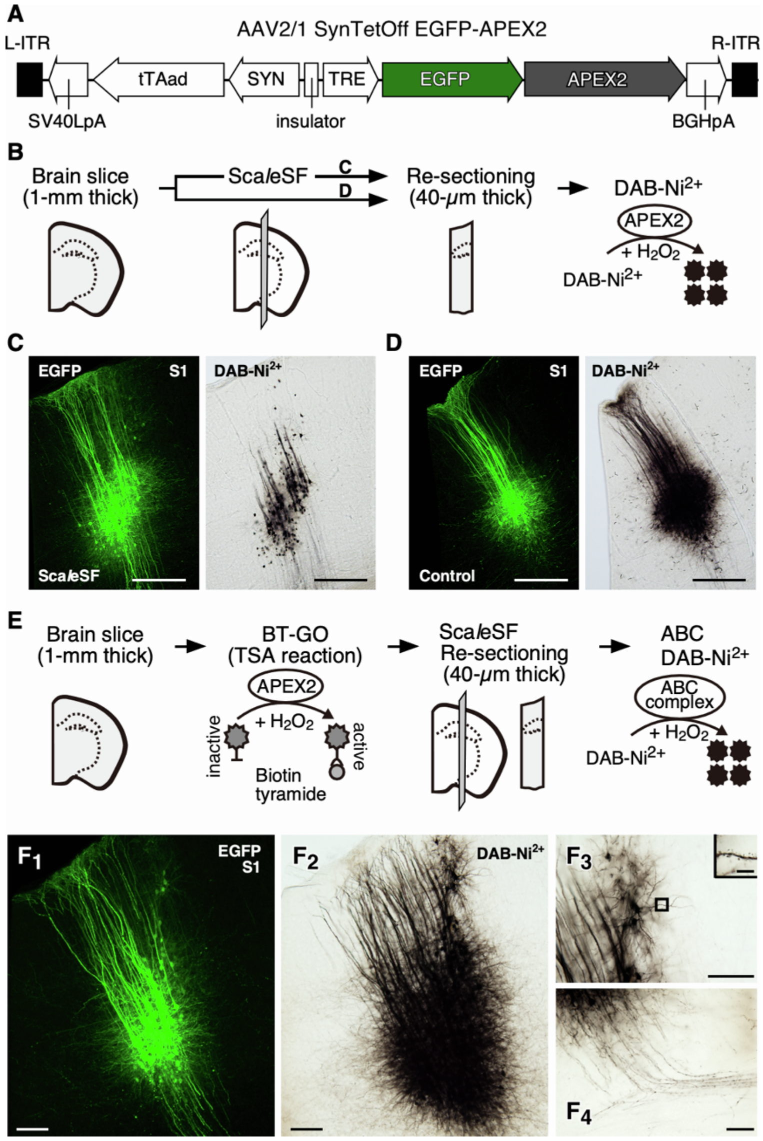

Figure S6. APEX2/BT-GO reaction enables correlated imaging of EGFP and DAB-Ni^{2+} polymers after Sca*l*eSF treatment. Related to Figure 5.

(A) The AAV2/1-SynTetOff-EGFP-APEX2 vector. BGHpA: polyadenylation signal derived from the bovine growth hormone gene, ITR: inverted terminal repeat, SV40LpA: polyadenylation signal of Simian virus 40 late, SYN: human synapsin I promoter, TRE: tetracycline-responsive element, tTAad: an improved version of a tetracycline-controlled transactivator.

(B) A schematic diagram of DAB-Ni^{2+} labeling with APEX2. Brains are fixed with 4% PFA containing 0.2% GA.

(C and D) DAB-Ni^{2+} labeling with APEX2 in mouse brain sections prepared from brain slices cleared with Sca*l*eSF (C, n = 6 injection sites from 3 mice) or stored in PBS(–) (D, n = 7 injection sites from 4 mice). Correlated fluorescence (left) and bright-field (right) images in neurons

infected with the AAV vector. After imaging with CLSM, sections are developed in DAB-Ni^{2+} solution. Scale bar, 500 μm in fluorescence and bright-field images.

(E) A schematic diagram of APEX2/BT-GO reaction-mediated signal amplification. Prior to clearing brain slices with Sca*l*eSF, biotin molecules are deposited with TSA reaction using peroxidase activity of APEX2. The cleared slices are cut into 40-μm-thick sections, and the sections are processed for ABC/DAB-Ni^{2+} visualization. Brains are fixed with 4% PFA containing 0.2% GA.

(F) Correlated fluorescence (F_1) and bright-field (F_2) images in a section of a mouse cerebral cortex processed with APEX2/BT-GO reaction-mediated signal amplification (n = 7 injection sites from 4 mice). High magnification images of dendrites (F_3), dendritic spines (the inset in F_3), and axon fibers (F_4) in the bright-field image are also shown. Scale bars, 100 μm in (F_1 and F_2), 50 μm in (F_3 and F_4), and 5 μm in (the inset in F_3).

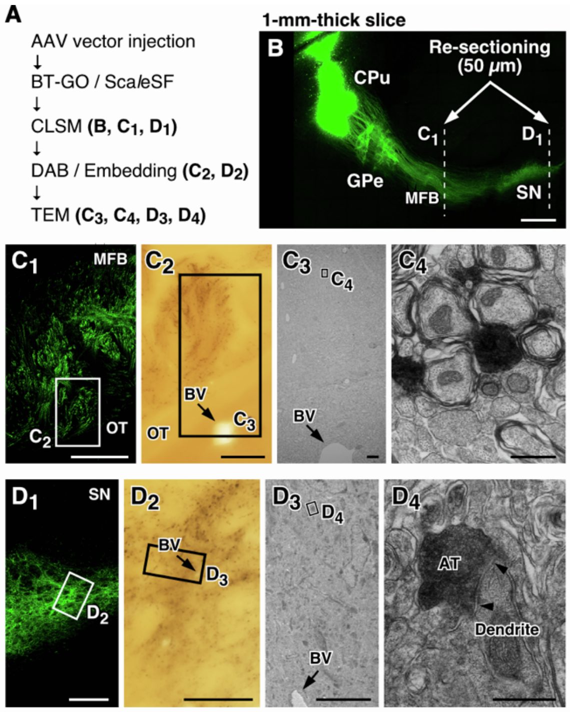

Figure S7. Multi-scale LM/EM neuronal imaging of the mouse striatonigral pathway. Related to Figure 5.

(A) The procedure of multi-scale LM/EM neuronal imaging of the mouse striatonigral pathway. Brains are fixed with 4% PFA containing 0.2% GA.

(B) A maximum intensity projection image of the striatonigral pathway labeled with the AAV2/1-SynTetOff-EGFP-APEX2 vector. A 1-mm-thick parasagittal brain slice is cleared with Sca*l*eSF four weeks after AAV injection into the CPu. Sections of 50-µm thickness are cut along dotted lines. Scale bar, 500 µm.

(C and D) Correlated fluorescence (C_1 and D_1), bright-field (C_2 and D_2), and TEM images (C_3, C_4, D_3, and D_4) at the level of MFB (C) and SN (D). AT: axon terminal, BV: blood vessels.

(C_1 and D_1) CLSM imaging. Scale bars, 200 µm.

(C_2 and D_2) DAB-Ni^{2+} labeling with APEX2/BT-GO reaction in the rectangle in (C_1) and (D_1). Scale bars, 50 µm.

(C_3 and D_3) A TEM image of the rectangle in (C_2) and (D_2). Scale bars, 10 µm.

(C_4 and D_4) A high magnification image of the rectangle in (C_3) and (D_3). Arrows in (C_2 and C_3) and (D_2 and D_3) indicate the identical blood vessel, respectively. Arrowheads in (D_4) point to a postsynaptic membrane. Scale bars, 500 nm.
